# Supplementary material for: brainlife.io: A decentralized and open source cloud platform to support neuroscience research
Source: ArXiv. 2023 Jun 8:arXiv:2306.02183v2. Preprint. [Version 2] (PMC10274934)
Supplement: Supplementary file 1 [file NIHPP2306.02183v2-supplement-1.pdf]

Supplementary material.

brainlife.io: A decentralized and open source cloud platform to support big data neuroscience research

Hayashi, S.*, Caron, B.*, Heinsfeld, A.S., Vinci-Booher, S., McPherson, B.C., Bullock, D., Berto, G., Niso, J.G., Hanekamp, S., Levitas, D., Kitchell, L., Leong, J., Silva, F. N., Koudoro, S., Willis, H., Jolly, J., Pisner, D., Zuidema, T., Kurzwaski, J., Mikellidou, K., Bussalb, A., Rorden, C., Victory, C., Bhatia, D., Aydogan, D.B., Yeh, F.C., Delogu, F., Guaje, J., Veraart, J., Fischer, J., Faskowitz, J., Chaumon, M., Fabrega, R., Hunt, D., McKee, S., Brown, S.T., Heyman, S., Iacovella, V., Mejia, A., Marinazzo, D., Craddock, C., Olivetti, E., Hanson, J., Avesani, P., Garyfallidis, E., Stanzione, D., Carson, J.P., Henschel, R., Hancock, D.Y., Stewart, C.A., Schnyer, D., Eke, D., Poldrack, R.A., George, N., Bridge, H., Sani, I., Freiwald, W., Puce, A., Port, N., and Pestilli, F.

Competing interests. The authors declare no competing financial interests.

Corresponding authors. Franco Pestilli pestilli@utexas.edu

Contribution. S.H. implemented the brainlife.io services. B.C. wrote the data analysis code, performed the large-scale experiments and prepared the figures and associated text. A.S.H. improved and implemented some of the services. F.J., C.C., C.C.A., D.H., D.S., D.P., L.K., J.L., C.R., F.N.S., H.W., J.J.,Z.T., K.J., S.K., N.A., V.C., B.D., A.D.B., F.D. G.J., S.H., provided assets. All authors edited the manuscript. F.P. invented, designed, and directed brainlife.io, and wrote the paper and designed all the experiments, and figures. *shared first authors contribution.

ABSTRACT

Neuroscience research has expanded dramatically over the past 30 years by advancing standardization and tool development to support rigor and transparency. Consequently, the complexity of the data pipeline has also increased, hindering access to FAIR data analysis to portions of the worldwide research community. *brainlife.io* was developed to reduce these burdens and democratize modern neuroscience research across institutions and career levels. Using community software and hardware infrastructure, the platform provides open-source data standardization, management, visualization, and processing and simplifies the data pipeline. brainlife.io automatically tracks the provenance history of thousands of data objects, supporting simplicity, efficiency, and transparency in neuroscience research. Here brainlife.io's technology and data services are described and evaluated for validity, reliability, reproducibility, replicability, and scientific utility. Using data from 4 modalities and 3,200 participants, we demonstrate that brainlife.io's services produce outputs that adhere to best practices in modern neuroscience research.

ABSTRACT 1

SUPPLEMENTAL RESULTS 2

Supplemental platform architecture 2

Supplemental Table 1: Platform services serving the brainlife.io platform. 5

Supplemental Table 2: Jupyter notebooks for analyses performed. 5

Supplemental Table 3: Preprocessing Apps used for the experiments. 7

Developing processing Apps for the platform 9

Using the platform 10

End-to-end reproducible scientific workflow 23

Supplemental platform evaluation 25

Supplemental platform utilization 25

Supplemental platform testing 27

Supplementary Table 4. Validity and reliability correlation tables. 33

Supplemental platform utility for scientific applications 34

Supplemental replication and generalization 35

Supplemental to detecting disease 36

Supplement to quality control at scale 37

Public services for promoting transparency and data gravity in neuroscience research. 39

Supplemental Table 5: Resources for data storage, archiving, and computational analysis. 39

SUPPLEMENTAL RESULTS

Supplemental platform architecture

brainlife.io is a composition of microservices, including authentication, preprocessing, warehousing, event handling, and auditing. Microservices are handled by a meta-orchestration workflow system, Amaretti (**Fig. S2a**,**b,** and **Table S1**). Amaretti can deploy computational jobs on high-performance compute clusters and cloud systems. Both jobs needed for platform operations and data analysis are handled by Amaretti. Amaretti is central to *brainlife.io*'s opportunistic computing approach, i.e., the ability to use donated storage or computing resources. Amaretti allows secure access to either clouds or supercomputers managing platform task scheduling, data transfer, and job submission and monitoring. Amaretti's core concepts are data- and resource awareness, i.e., data products or compute resources are specified as objects that the platform has explicit awareness of (e.g., the platform can dock datatypes, or compute resources; **Fig. S2b**). For example, users and resource managers can register a computing resource, making it available via *brainlife.io* either privately (to a specified set of users) or widely (to the entire platform users base). A variety of resource architectures and job submission systems have been tested and docked using Amaretti so far, including SLURM, PBS, OSG Engine, and CONDOR. Currently, Amaretti is hosted by a public cloud [1,2] and connected to major data centers (via access-ci.org; see **Fig. S2**) and commercial clouds.

Data processing on *brainlife.io* utilizes an object-oriented service model, based on micro workflows. Apps and datatypes work together to allow smart docking and awareness (**Fig. S2a, b,** and **c; Fig. S2b**). Apps are modular, composable processing units comprising either full pipelines [3–23] or small steps within a larger data-processing workflow. Apps are written in a variety of languages following a lightweight specification (github.com/brainlife/abcd-spec) and using containerization technology [24,25]. Containerization allows deployment on various compute resource architectures (hub.docker.com/u/brainlife). Apps code is hosted on github.com. Code must be first registered on *brainlife.io* in order to become an App. An App registration process guides

developers to map both input and output data objects to *brainlife.io* datatypes via a graphical interface. For security reasons, platform administrator approval is required to allow Apps on compute resources. A DOI [26–28] is issued for registered Apps to support scientific transparency and credit assignment to developers [29–39]. App specification requires developers to provide an informative readme file on GitHub with proper citations to software and funding used for the App (**Fig. S3a**). After registration, platform users can access Apps via a graphical (GUI) or command line interface (CLI). Apps can run on multiple resources, and Amaretti has methods for matching Apps to resources based on criteria such as geolocation, performance profiles, and resource queue length.

Apps on *brainlife.io* are data-aware and can automatically identify datasets, dock them or send them elsewhere for processing. This is because data objects are stored using predefined formats —datatypes. Datatypes allow App concatenation and automated pipelining (**Fig. 2c**; brainlife.io/datatypes). Datatypes comprise collections of files and folders organized into *.tar* archives to limit the number of inodes needed for storage. A platform-side datatype validation service (github.com/brainlife/?q=validator-) assures that datatypes comply with their definition. Data are physically stored using S3-like storage buckets organized following the pattern: `<s3bucketName>/<projectID>/<datasetID>.tar` Buckets can live in multiple geolocations, so as to help with international requirements [40] (**Fig. 2b**). Datatypes comply with BIDS[41] (if the standard is defined for the data objects).

Data management is centered around Projects and supported by a databasing and warehousing system (github.com/brainlife/warehouse). Projects are the "one-stop-shop" for data management, processing, analysis, visualization, and publication (**Fig. S3b**). Projects are created independently by users and are private by default, but can be made public within the *brainlife.io* platform. Projects provide stratified access control mechanisms, and data user agreements can be added to the landing page (see **Video S1**). A project can be populated with data using several options (**Fig. 2d**). Major archives and data repositories are docked by brainlife.io[42] (see **Fig. 2b**). Noticeable examples are OpenNeuro.org [43], and the Nathan-Kline data-sharing project [44–46]. Datasets can be seamlessly imported into *brainlife.io* Projects via the portal brainlife.io/datasets (see **Video S2** and **Video S3**). MRI, EEG, and MEG files (e.g., DICOM, .fif, .ctf) can also be uploaded directly using either a GUI (**Video S4**) or CLI (**Video S5**). A DICOM to BIDS conversion service has also been developed for MRI data standardization and importing into Proejcts (brainlife.io/ezbids; see **Table S1** and **Video S6**).

The data workflow in *brainlife.io* reduces the complexity of the neuroimaging processing pipeline into two steps akin to the MapReduce algorithm [47]. An initial *map step* preprocesses data objects asynchronously, is parallel using Apps, so as to extract features of interest, such as functional or white matter maps, or time series data (**Fig. 2d**). During the *map step*, datatypes and Apps are synchronized and moved across available compute resources automatically, as optimized by Amaretti. Apps process data objects automatically and in parallel across study participants in a Project. A dedicated web interface exists to explore sequences of Apps and optimize the parameters for each data set (**Video S7**). In addition, App sequences can be composed using a Pipeline builder interface (**Video S8**).

The *map step* is followed by a *reduce step*. Features extracted using Apps are synchronized, brought together, and made available to Jupyter notebooks[48,49] for statistical analysis and to generate figures for scientific articles (all figures in the following sections of this paper are available in Jupyter notebooks, see **Table S2**). App developers can identify datatypes as "statistical features." Datatypes that are made accessible via Jupyter Lab interfaces hosted inside a Project (**Fig. 2d** left, **Fig. S2a**, and **Video S9**). The statistical features are automatically organized by *brainlife.io* into *Tidy data* formats [50] (*.tsv* and *.json*; **Fig. 2d**) and can be exported using the *pybrainlife* Python module (https://pypi.org/project/pybrainlife/). Jupyter Lab records are tracked for reproducibility and allow data analysis in R, Python, or Octave [48,49].

The full data workflow (from import to preprocessing to analysis) makes possible the unification of large volumes of diverse neuroimaging datatypes into simpler sets of features organized into *Tidy data* structures [50] (**Fig. S3c**). The platform provides a variety of methods to visualize data, which aids in performing quality assurance, identifying mistakes, and repeating the processing when needed. Community-developed visualizers are served on the cloud side using docker containers (see **Table S1**), and six new web visualizers have been developed (**Table S1** and **Video S7**).

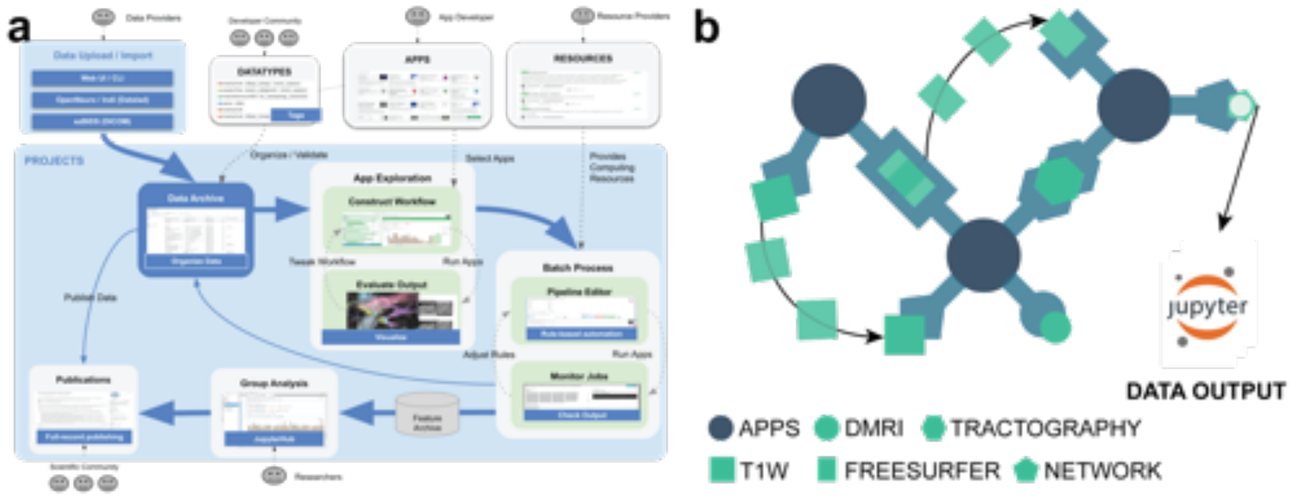

Supplemental Figure 2ab Brainlife architecture and system components. a. Illustrative flowchart of the multi-faceted architecture of brainlife.io. Data providers, developer community (datatypes), app developers, and resources providers. Data providers upload their data to brainlife.io Projects via web UI/CLI, OpenNeuro or DataLad, or ezBIDS. These data are then stored and organized into Projects organized as "datatypes" based on specifications from the developer community. App developers develop self-contained Apps that can then be used to construct workflows from data inputs to final statistical products. Visualizers and QA measures allow for better construction of workflows to ensure the highest statistical quality and fidelity. These Apps run on compute resources provided by many resource providers, including HPC and cloud providers. Once workflows have been optimized, batch processing can be performed via Pipeline rules. These rules automatically track progress, including app completion and failures. Once statistical features of interest have been extracted, they are pushed to the warehouse into JupyterHub, where collaborators can work together on analyzing the data for the entire project. Upon completion, brainlife.io facilitates the publishing of the data and workflows via a Publications mechanism, where data and workflow information are reorganized for easier download and dissemination and given a digital object identifier (DOI) for the scientific community. **b.** An illustrative example of generating a network datatype on brainlife.io starting from structural (T1w) and diffusion (DWI) data on brainlife.io. Datatypes get docked as inputs into apps, from which either modified versions of the input are returned as output or entirely new datatypes are generated. Datatypes can be shared amongst other apps in the workflow (*arrows*) allowing for the chaining together of Apps into entire workflows. The outputs of this workflow can then be pushed to Jupyter Notebooks for statistical analysis.

The ABCD specification and brainlife.io Apps. The Application for Big Computational Data (ABCD; github.com/brainlife/abcd-spec) is a lightweight, specification proprietary to *brainlife.io* that enables App developers and resource managers to establish programming interfaces, to facilitate the integration of applications with the job scheduling systems (PBS, CONDOR, SLURM, etc) associated with a resource. The interfaces encompass the "`start`" entry point, used to initiate an service, the "`status`" interface, invoked to track the progress of service's job status and the "`stop`" interface, invoked to conclude the execution of service.

Amaretti decentralized resource awareness and prioritization. Amaretti is a meta-orchestration system able to run any App or service published on GitHub and conforming with the ABCD specification. Amaretti is "meta" in the sense that it make use of the underlying batch-scheduler (job-orchestration) mechanism already existing in computing resources. Amaretti has the ability to run services distributedly on multiple computing resources. In the event that a particular service is enabled on multiple resources, Amaretti utilizes a selection mechanism to choose the optimal resource. For example, a data processing workflow can consists of multiple steps, each implemented in a *brainlife.io* App or service. Amaretti allows sending each step in a sequence of processing steps on a different resource. The same step may be sent to different resources everytime it is requested. The outputs resulting from each step are then synchronized after execution is completed. If a user has access to multiple resources on which an App or a service can be executed, Amaretti decides selects a resource using a series of heuristics. At runtime, Amaretti computes the final resource and decides which resource to use for a service by using the following rules:

1. *Resources scoring.* Resource managers enable Apps or services on a resource. The manager can define a default score for the App, the higher score the more likely that the resource will be selected to execute a service. Find the default score configured for the resource. If not configured, the resource is disqualified from being used (resource managers must give explicit permission to run the App)

2. *Inter-resources data transfer minimization.* For each App data dependency, the score is incremented by 5 if the resource is used to run the Apps that generate the prerequisite data. This increases the likelihood of reusing the same resource where App runs produced data that is already available on the resource. This approach mitigates data transfer.
3. *Exclusive resource ownership criteria.* An additional ten points are given to a resource if the user possesses exclusive ownership of the resource. Users can define resources only assigned to them. In such case, rather than utilizing a shared resource, it is advantageous to use the private resource.
4. *Preferred resource ownership criteria.* An increment of fifteen points is added to the score when the resource is designated as the preferred resource to use, as stipulated by the user that submitted the App execution request.
5. *Public resource avoidance.* A project can be configured by users to abstain from using public computing resources. Public resources become ineligible for consideration if the App execution request originates from such a project.
6. *Connection failure.* A resources is disqualified if the resource monitor service detects a connection or server failure.

The resource with the highest score is chosen to execute the task, and a report detailing the rationale behind the resource's selection is added to a file within the service working directory

Tasks. Tasks are the atomic unit of computational work executed on various compute resources. Examples of Tasks are, a job for batch systems, or a vanilla process running on a vanilla VM. Amaretti keeps track of tasks by assigning each one of them a unique process ID.

Service. Any ABCD-compliant GitHub repository is a service for Amaretti. Apps are Amaretti services. When users or the platform submit a task Amaretti retrieves the code service from GitHub. For example, if the user requests to run the Task specified by github.com/brainlife/app-life App, Amaretti will retrieve the code from GitHub, create a copy of the App for that task on a chosen resource and also move.

(Workflow) Instance. Amaretti provides DAG workflow capability by establishing dependencies between tasks. Tasks that depend on parent tasks will simply wait for those parent tasks to complete. All Amaretti tasks belong to a workflow instance (or instance for short).

Resource. Resource is a remote computing resource where Amaretti can securely connect and set up the App execution through the ABCD interface. The resource can be a single computer, a head node of a large high-performance computing cluster, or a submit node for high-throughput computing clusters. The code for the brainlife.io platform is available at https://github.com/brainlife/.

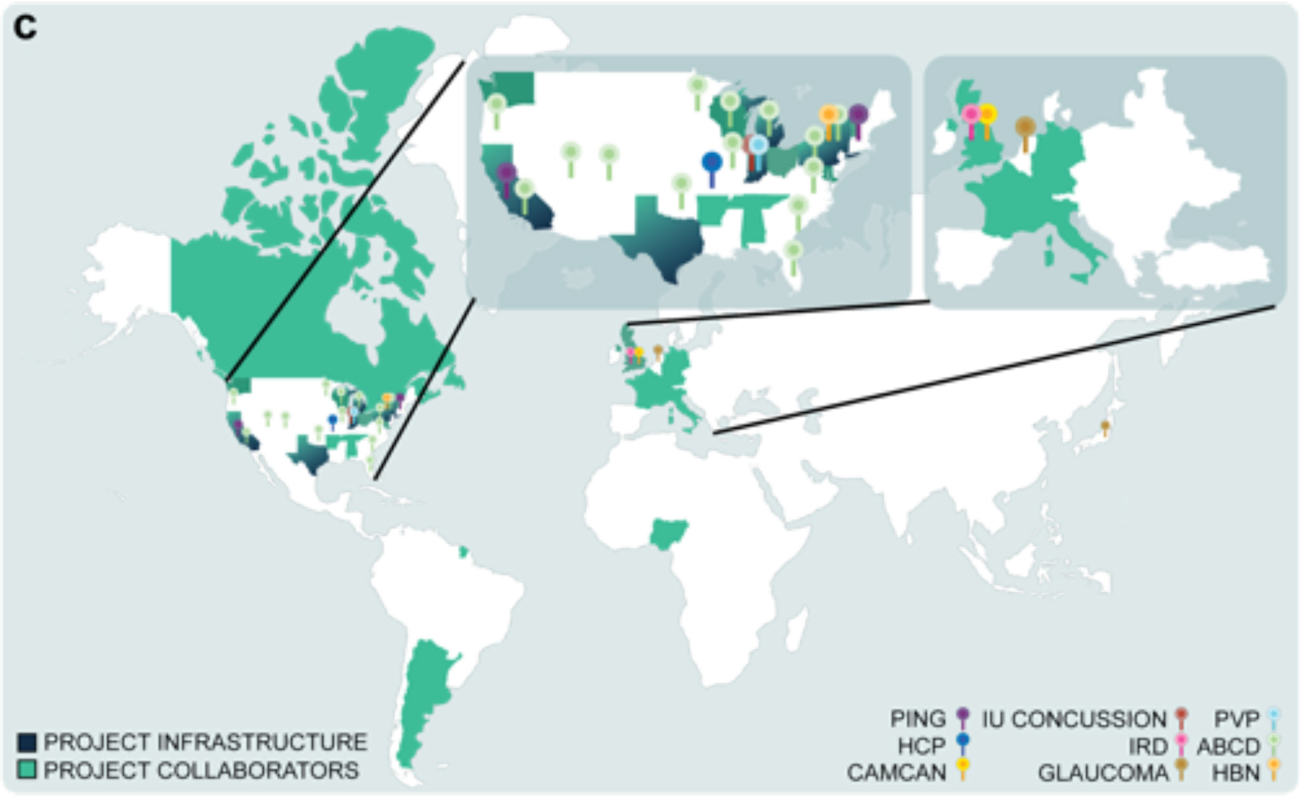

Supplemental Figure 2c. System components. c. Map the locations of critical facets of this research, including project infrastructure (i.e. compute resources), collaborators, and data sources. As the United States and Europe are home to many of the infrastructural resources, collaborators, and data sources, more details for these regions are provided (*insets*).

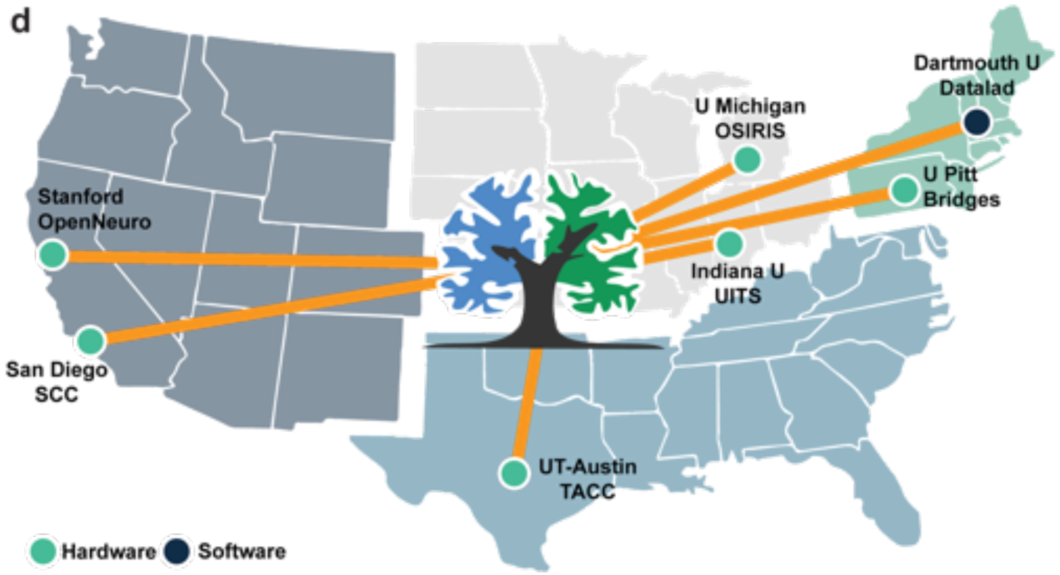

Supplemental Figure 2d. *brainlife.io* infrastructure geolocation (2023). d. Map of the locations of critical hubs for *brainlife.io*

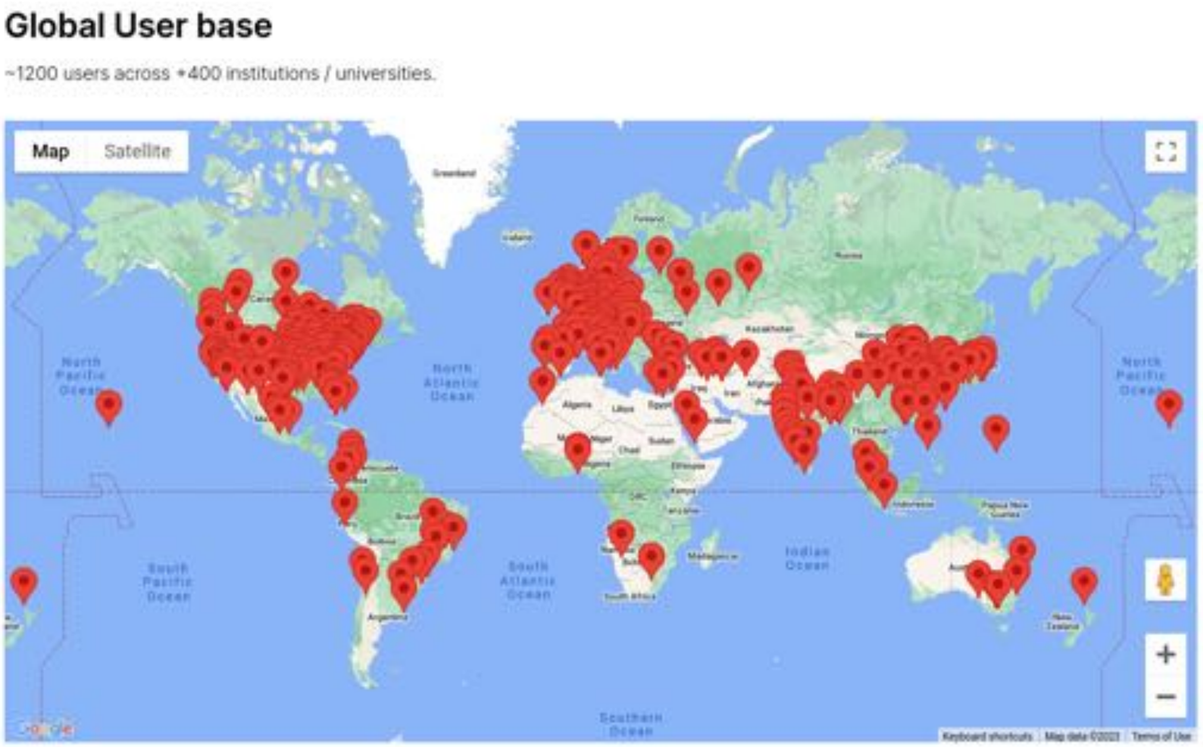

Supplemental Figure 2e. brainlife.io user account geolocations. e. Map of the locations of the users that created an account and accessed *brainlife.io.* This map is a proxy to the level of attention the platform achieved worldwide.

Supplemental Table 1: Platform services serving the brainlife.io platform.

The brainlife.io platform is an incorporation of many individual services working in concert to increase the efficiency of neuroimaging analyses on the scale of thousands of participants and brain datasets. In addition to our efforts to compile a list of currently available services provided by the greater scientific community, below we provide a list of the many platform services that combine to make the brainlife.io platform (**Table S1)**. Because brainlife.io is an open platform for neuroscientific investigations, we provide the individual URLs pointing to the code base of the individual services of the platform.

Supplemental Table 2: Jupyter notebooks for analyses performed.

The code used to analyze the thousands of datasets processed in this manuscript is openly accessible on GitHub.com. Below we provide a list of the jupyter notebooks for performing the analyses outlined previously (**Table S2**). For this, we provide the jupyter notebook name and the GitHub URL for the respective notebook. Within each notebook, we describe the neuroimaging topic the notebook covers, including structural morphometry (i.e. cortical thickness, surface, area, volume), diffusion profilometry, structural connectivity, functional connectivity, functional gradients, MEEG, and optical coherence tomography (OCT). These notebooks were used to summarize data for different measures and many individual analyses and figures outlined previously. The goal of these notebooks is to document enough information for new users to re-use the notebooks for their own analyses on their own datasets. These notebooks are freely available for use by the greater scientific community.

Supplemental Table 3: Preprocessing Apps used for the experiments.

In addition to providing documentation to the code servicing brainlife.io, we openly release the App code for each App used to analyze the thousands of datasets processed in this manuscript. Below we provide a list of the Apps used for performing the analyses outlined previously (**Table S3)**. For this, we provide the App name listed on brainlife.io, the digital-object identifier (DOI) automatically assigned to each app, and the GitHub Repository where the code for the App resides. The goal of this is to increase the transparency of the processing steps performed in this investigation, and for researchers to validate and incorporate into their currently existing workflows.

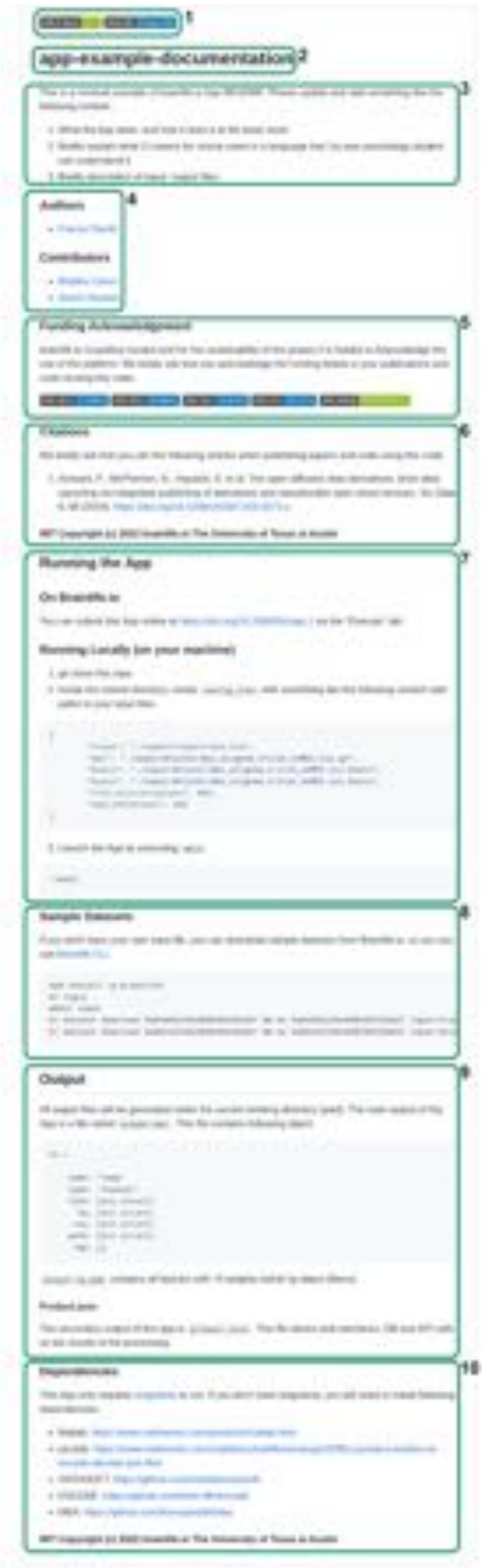

Supplemental Figure 3a. Brainlife App Github template. 1. App DOI and ABCD specification. 2. App name. 3. Description of the App. 4. Authors and contributors. 5. Funding Acknowledgement. 6. Citations. 7. Instructions for running the app locally, including how to set up the config.json file containing all of the important information for the App including inputs and configuration parameters. 8. Example datasets that can be downloaded to test the app locally. 9. The outputs for the App. 10. The software dependencies subserving the App.

Developing processing Apps for the platform

Here we describe the requirements for developing Apps on the platform. Despite the over 500 apps currently available on the platform, there still exist possibilities for researchers to develop their own processing Apps for performing specific steps that might not already exist on the platform.

The development process for Apps has been streamlined in order to make it as intuitive as possible. Specifically, each App has a set of requirements necessary for the App to be used on the platform. The most important of these requirements involves the creation of a README file outlining all of the important information needed to describe the contents of an App. On Github, we have developed a set of App README templates for App developers to use (**Fig. S3a**). On the README file, the user must provide information regarding the brainlife.io App DOI and the ABCD specification. In addition, they must also document the app name and a description of what steps the App performs.

Users can also provide information regarding specific authors, coauthors, funding sources, and literature citations in order to provide proper credits for the development of the App. Following these descriptive details, the README should also provide information regarding the usage of the App both on brainlife.io and on local workstations, including descriptions of the inputs, outputs, and software library dependencies of the App. These descriptions found in the README increase the transparency of the App in order to increase the findability and usability of the App.

Using the platform

Here, we describe the user interface of the platform to help introduce the visual interfaces developed as part of the project. These steps will be described in order of how they would be implemented by a typical researcher designing their own set of experiments using the platform. In addition to visual and text descriptions, we also provide a series of videos documenting each step of the process.

Upon creation of a brainlife.io account, a researcher will first set up a Project within which all of the data processing, storing, and organization will occur (**Fig. S3b; Video S1**). Once their Project is created, users can then update and assign details to the project, including a description of the project, access control to the project, a project README file describing specific information about the project in a machine-readable format, information regarding each participant in the study, and even limit which computing resources the Project will use to process the data.

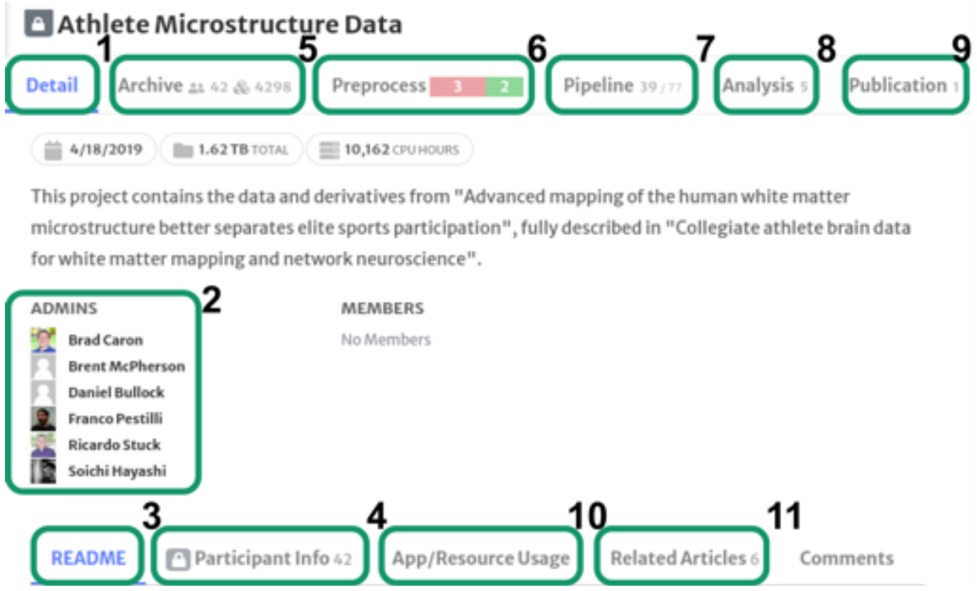

Supplemental Figure 3b. Brainlife project landing page. 1. Detail tab containing all of the important details and information describing the Project. 2. Users can add Admins and members for proper project governance. **3.** Projects can have README descriptions, like those on GitHub, to describe important details of the project in a Markdown format. **4.** Participant Info contains tables of demographic information that may be helpful for performing an analysis. This is set and defined by the Administrators of the Project. **5.** Archive tab is where all of the stored files in the form of brainlife datatypes can be found. **6.** The Preprocess tab is where jobs can be launched and monitored. **7.** Pipelines allow the investigator to batch process all of the participants in their project for each App they need to run. **8.** Once statistical features have been extracted, Administrators can access Jupyter Notebooks within the Analysis tab to perform their statistical investigations across all of the participants in the project. **9.** Once the investigators are completed the investigation, they can use the Publication tab to efficiently publish their data and the analysis workflows on brainlife.io. **10.** Whenever a job launches, the App/Resource Usage tab is automatically updated in order to provide provenance tracking of what and where the data processing was performed. **11.** Brainlife.io will search keywords in your project with previously published studies to identify any related articles to your investigation in the Related Articles tab.

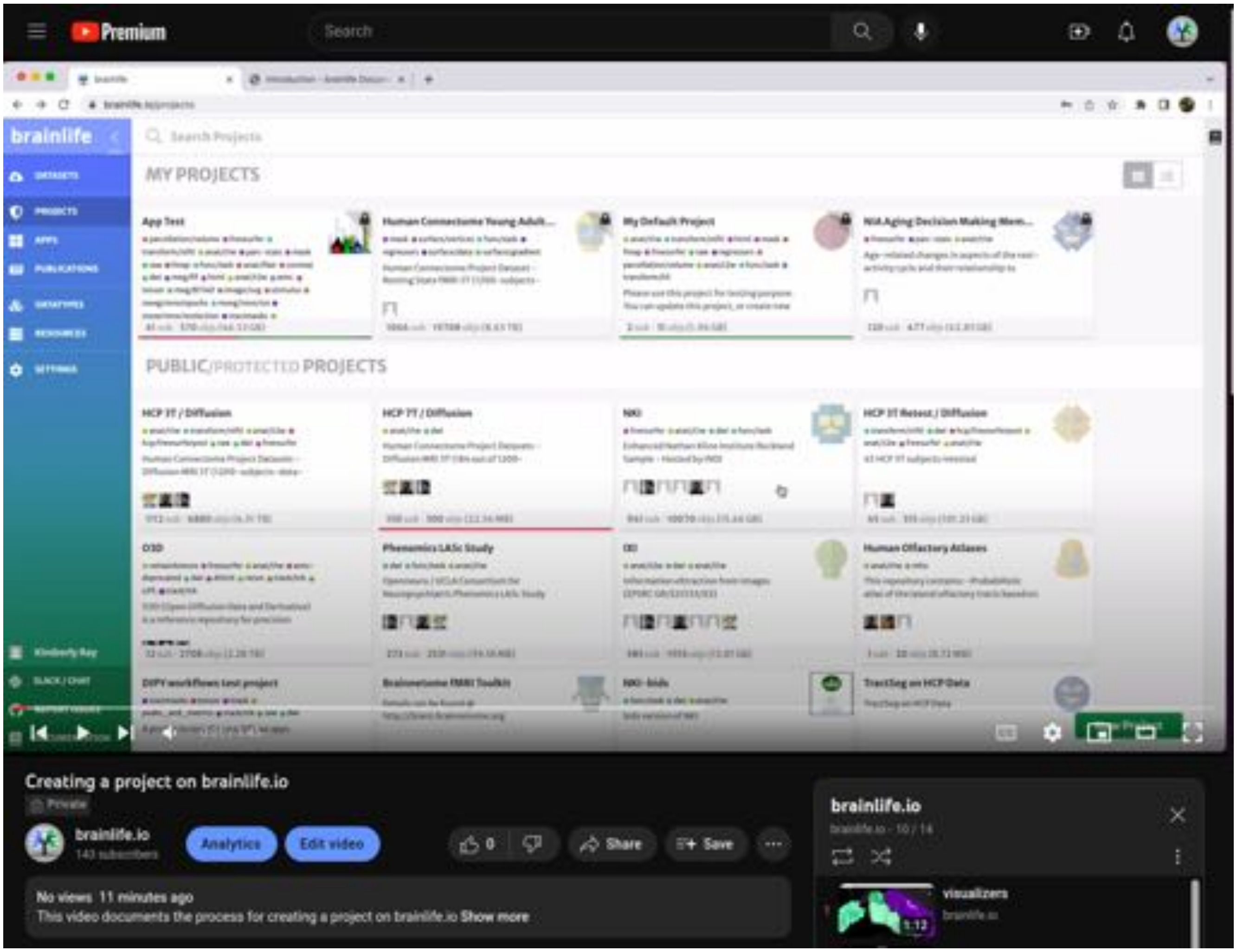

Supplemental Video 1. A video documenting the process of creating a project on brainlife.io, including updating access control and participant information. https://youtu.be/P2kz6E53nlo

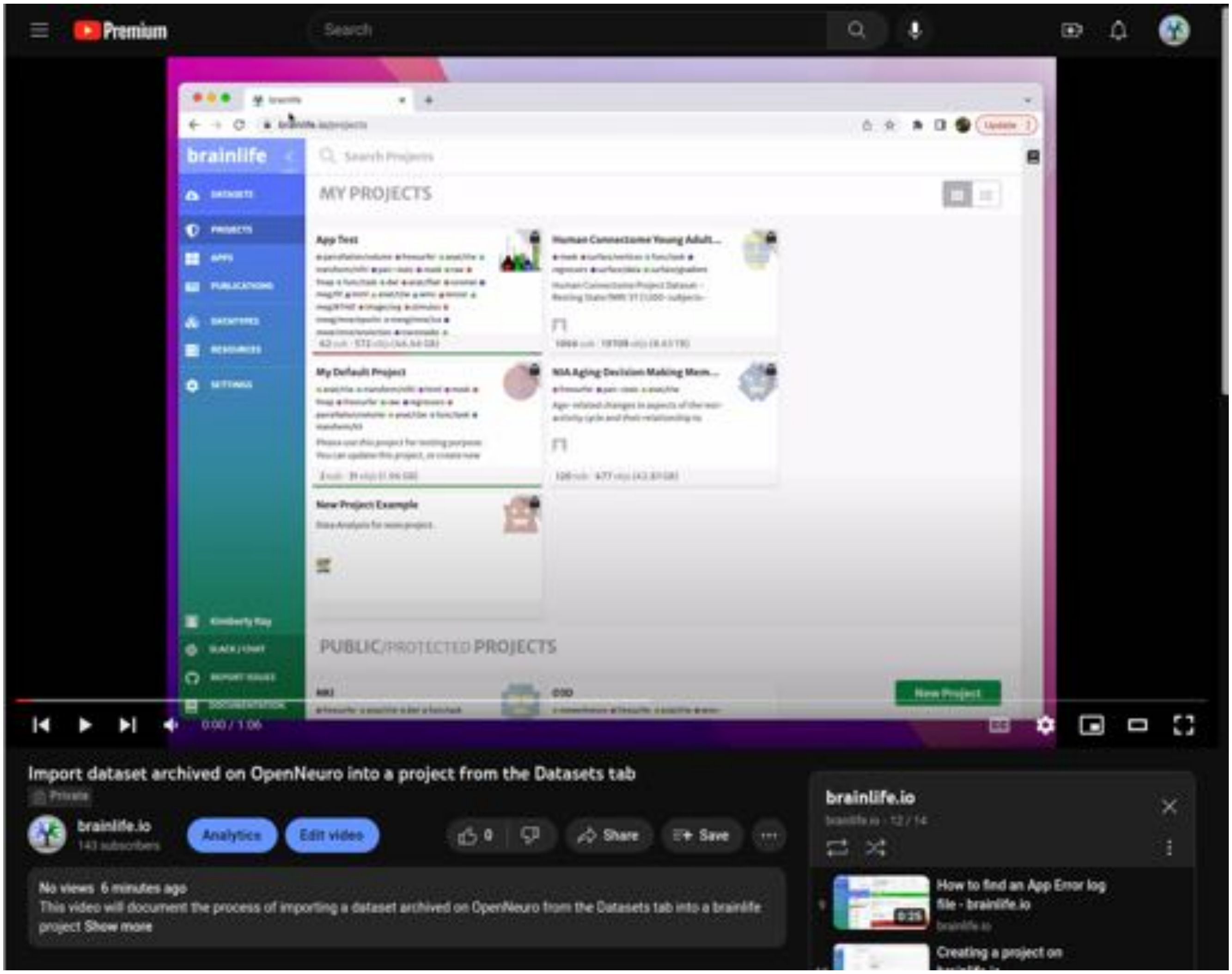

Supplemental Video 2. A video documenting the process of pulling datasets from the 'datasets' tab into a Project on brainlife.io. https://youtu.be/N3UXteQ3tu8

Once this information is defined, users are then ready to either import raw datasets they collected or pull datasets that have been openly released. For openly released datasets, users have a variety of options to pull data from including other projects (**Video S2**), or projects hosted on OpenNeuro (**Video S3**). In a similar fashion, users have a variety of options for uploading any newly collected datasets including a built-in GUI (**Video S4**), a CLI (**Video S5**), or through a newly developed sister technology for automated converting of raw scanner data into BIDS-standardized data files known as ezBIDS (**Video S6**). Each of these methods provide a streamlined, efficient way to import data into a new project for future processing and analysis.

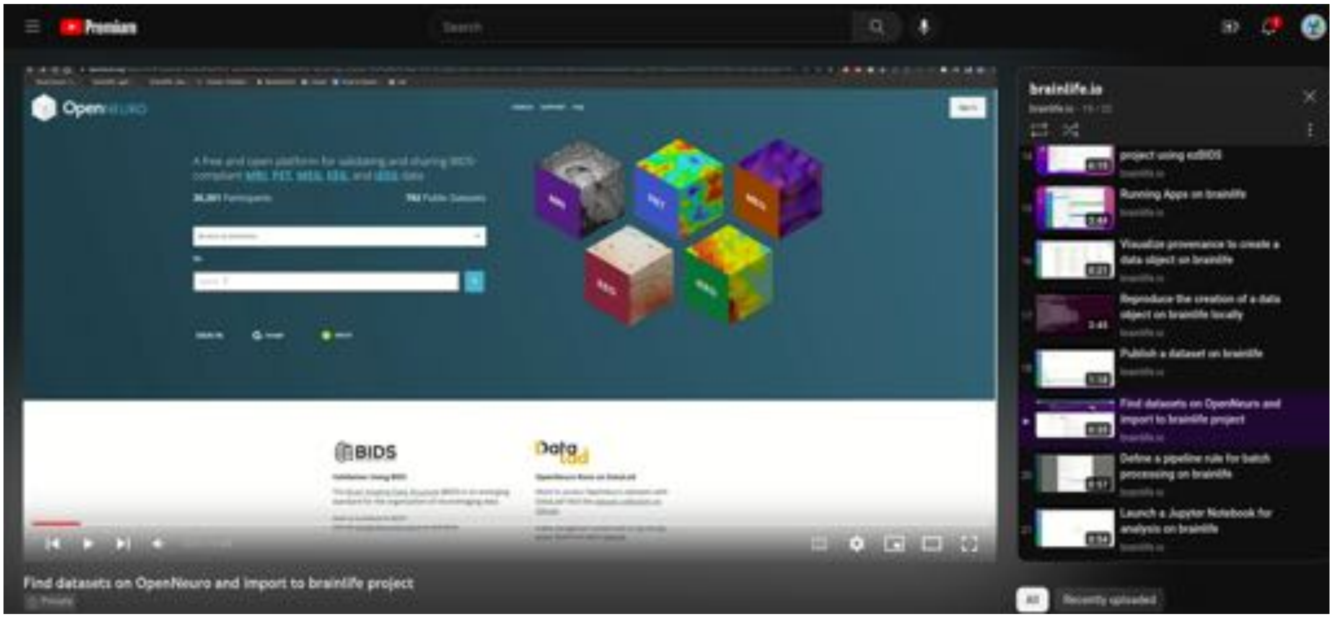

Supplemental Video 3. A video documenting the process of pulling data from OpenNeuro into a Project on brainlife.io. https://youtu.be/OZQyR9jLwYo

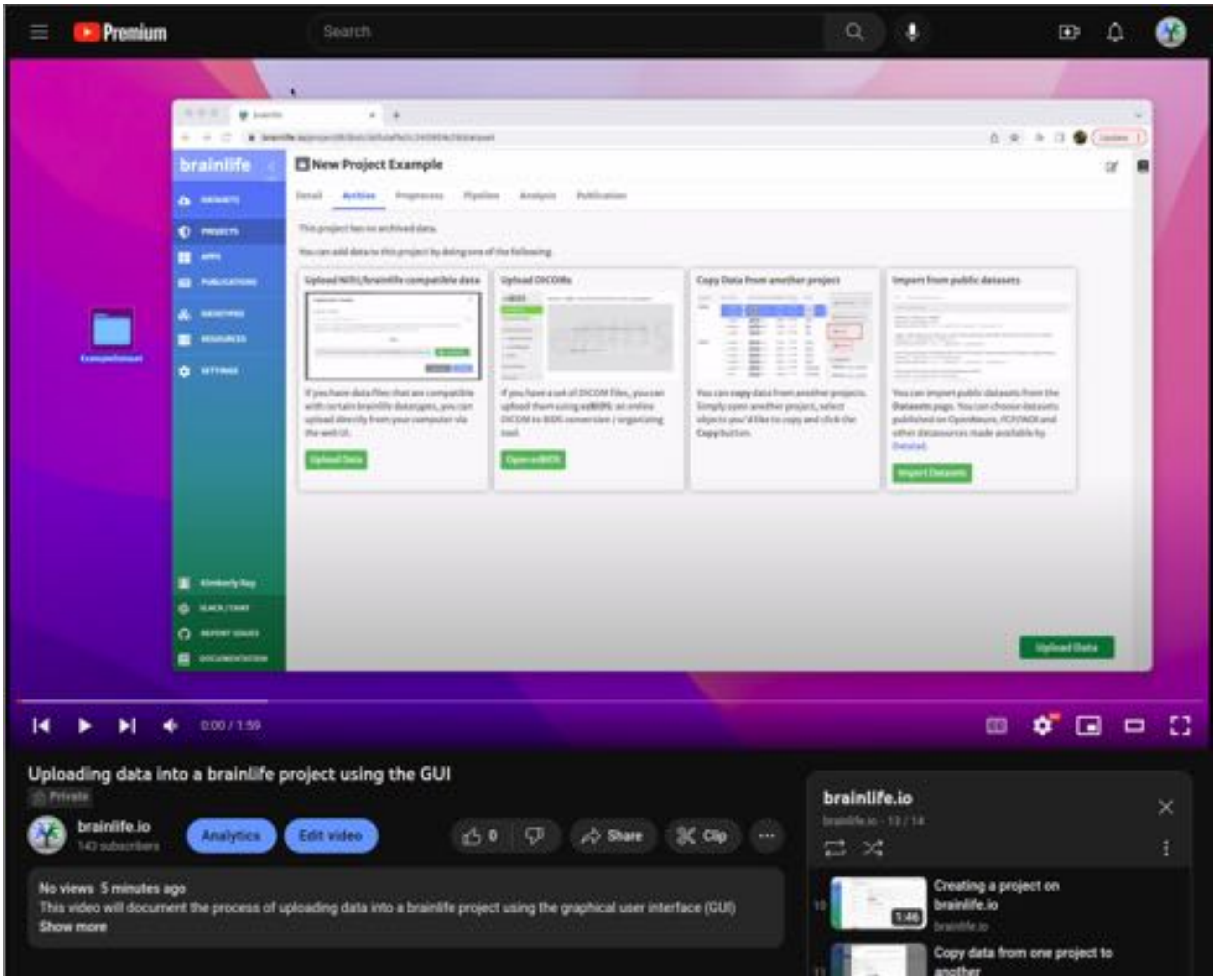

Supplemental Video 4. A video documenting the process of uploading data to a brainlife project using the graphical user interface (GUI) directly via the browser. https://youtu.be/5RGo_jY4Oqc

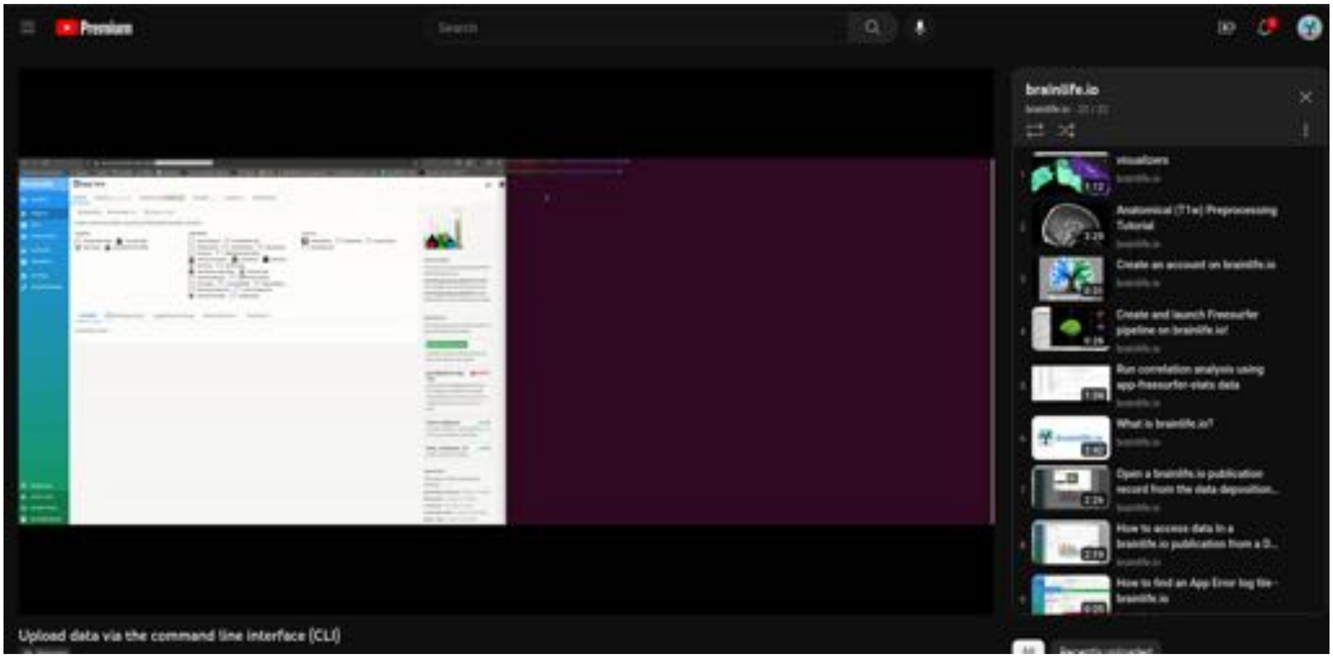

Supplemental Video 5. A video documenting the process of uploading data to a brainlife project using *brainlife.io*'s Command Line Interface (CLI). https://youtu.be/PUTLXJJSBqQ

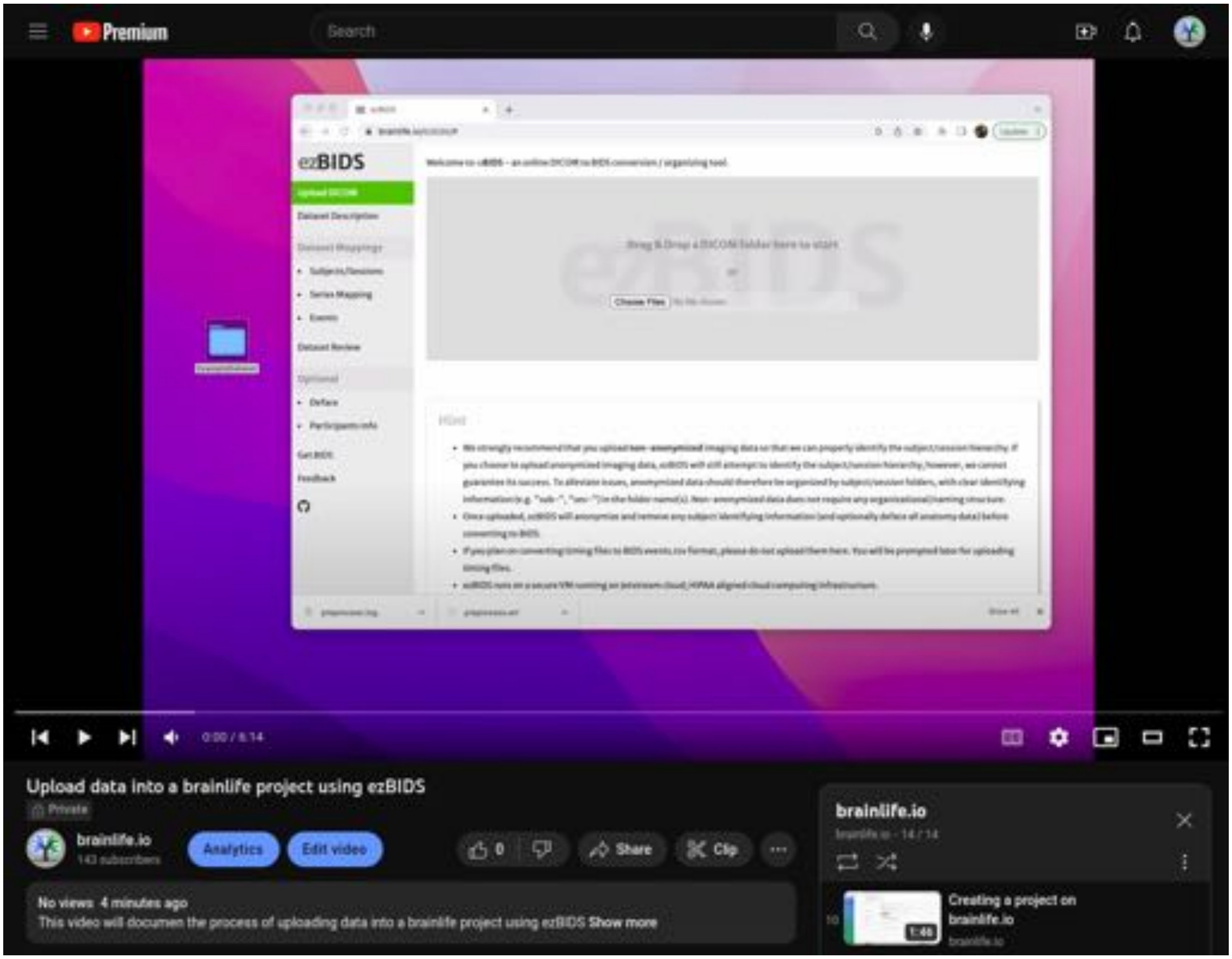

Supplemental Video 6. A video documenting the process of uploading data to a *brainlife.io* Project using the DICOM to BIDS converter brainlife.io/ezBIDS. https://youtu.be/KvhIHxzHsl4

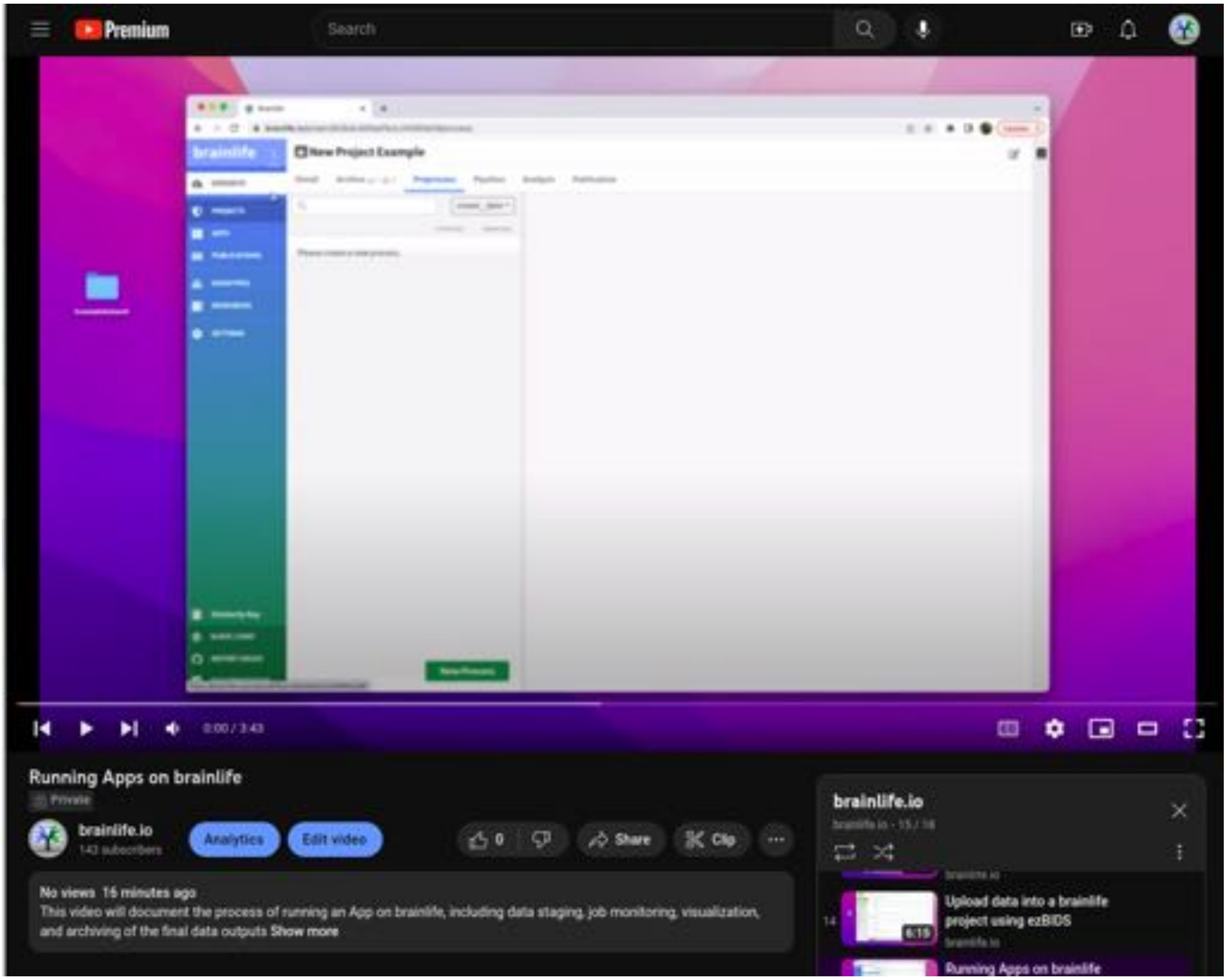

Supplemental Video 7. A video documenting the process of running an App on brainlife.io, including data staging, job monitoring, visualization, and archiving of the final data outputs. https://youtu.be/43yhZ1k6icQ

Upon importing data into a Project, users can directly interact with the data stored in the Archive tab of the project in multiple ways. First, users can select a data object and visualize the data object using one of the many built-in visualization services for that specific datatype. More importantly, users can then "stage" or move the data from Archive into the Preprocess tab, from which users can select and launch any of the over 400 available Apps (**Video S7**). Because Apps on brainlife are "data aware", users will only be presented with the Apps that take in the staged datatypes that they are designed to work with as inputs ultimately reducing the potential for user error. From the Preprocess tab, users can monitor the status of the App, interact with the data files generated during the App, and visualize the outputs. Once the user is satisfied with the outputs, data objects can be stored back into the Archive tab directly from the Preprocess tab.

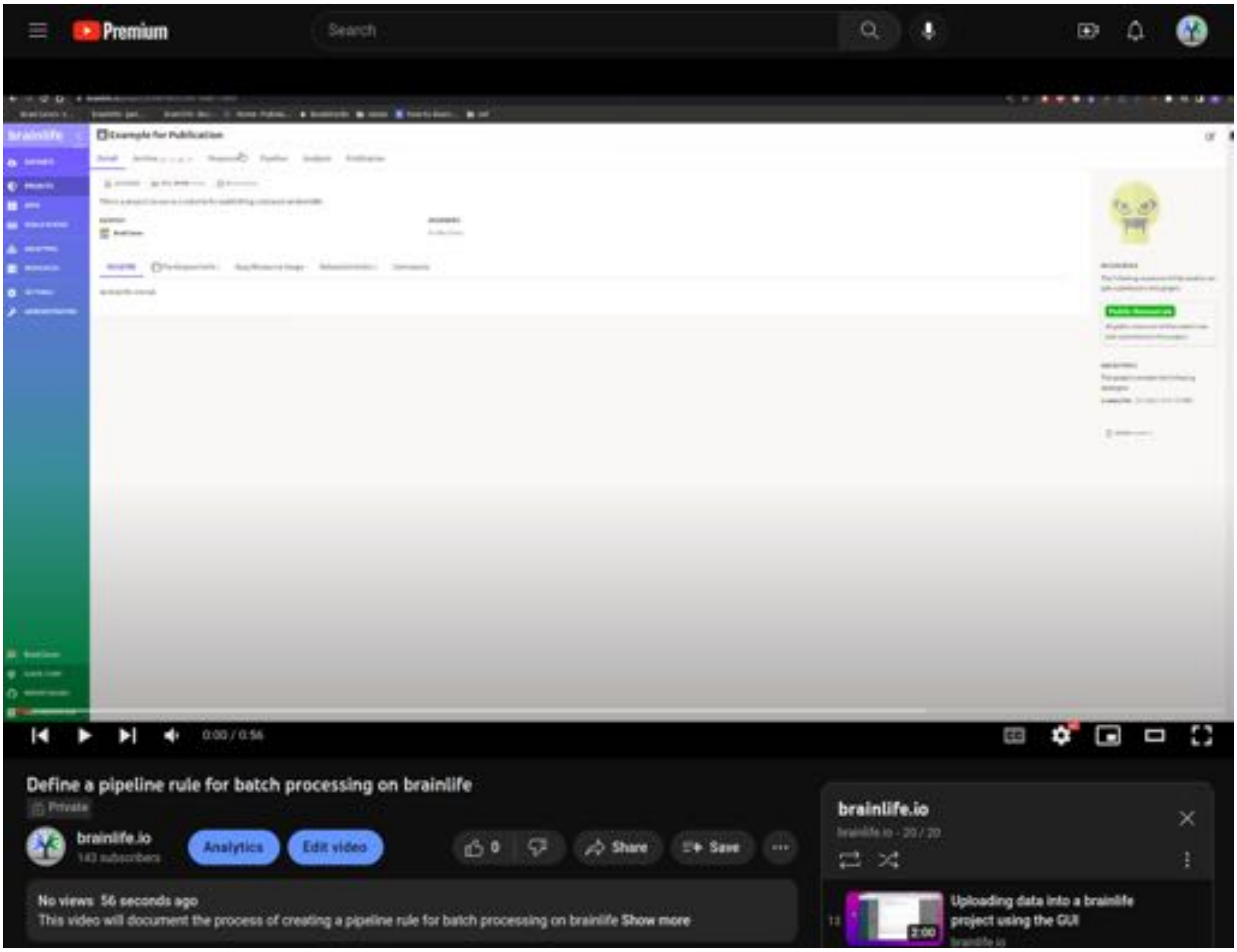

Supplemental Video 8. A video documenting the process of defining a Pipeline rule on brainlife.io to perform batch processing. https://youtu.be/1CSdsf8czL8

This process for running an App is useful under testing circumstances, but may not be appropriate for batch processing of a large number of participants. To facilitate this, users can define Pipeline rules via the Pipeline tab (**Video S8**). Within these rules, users specify the inputs including which data objects from the Archive to include or exclude, the configuration parameters required by the App, and the archiving of output objects back into the Archive. Upon launching a Pipeline rule, Amaretti will automatically stage all of the data that matches the input criteria, identify the most appropriate compute resource for running the process, and archive the output data objects back into the project Archive for storage. Outputs from one Pipeline can then be set as inputs to another Pipeline, allowing for the chaining of Apps to develop the overall processing workflow required to get from raw data to the final statistical features of interest needed for statistical analysis.

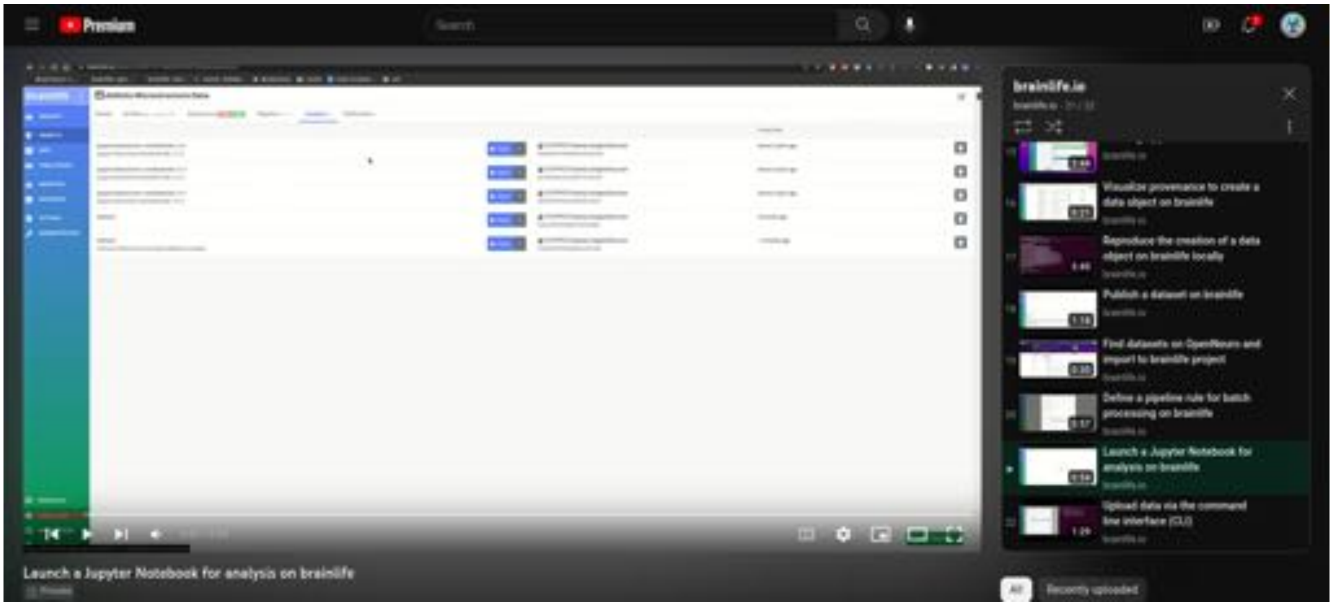

Supplemental Video 9. A video documenting the process of launching a Jupyter Notebook for performing statistical analyses within a brainlife project. https://youtu.be/tJW6374BcpQ

Once these statistical features of interest are extracted, users can then analyze them directly on the platform via the Jupyter Notebooks provided by brainlife.io (**Video S9**). To facilitate this, a certain subset of all datatypes that correspond to statistical features of interest are stored in a secondary warehouse, which can be directly loaded via the Jupyter Notebooks. This ultimately reduces the number of potential data objects and storage size of the objects required by brainlife.io to move into the Notebooks, ultimately making the process more efficient for users. Common subsets of functions, including those useful for loading data into the Notebooks, have been packaged into a Python package *pybrainlife* that can be imported directly into the Notebooks and used to load and compile an entire study's worth of statistical features. Upon completion of the analyses, these Notebooks can be directly published and/or pushed to Github in order to increase the scientific transparency of the project.

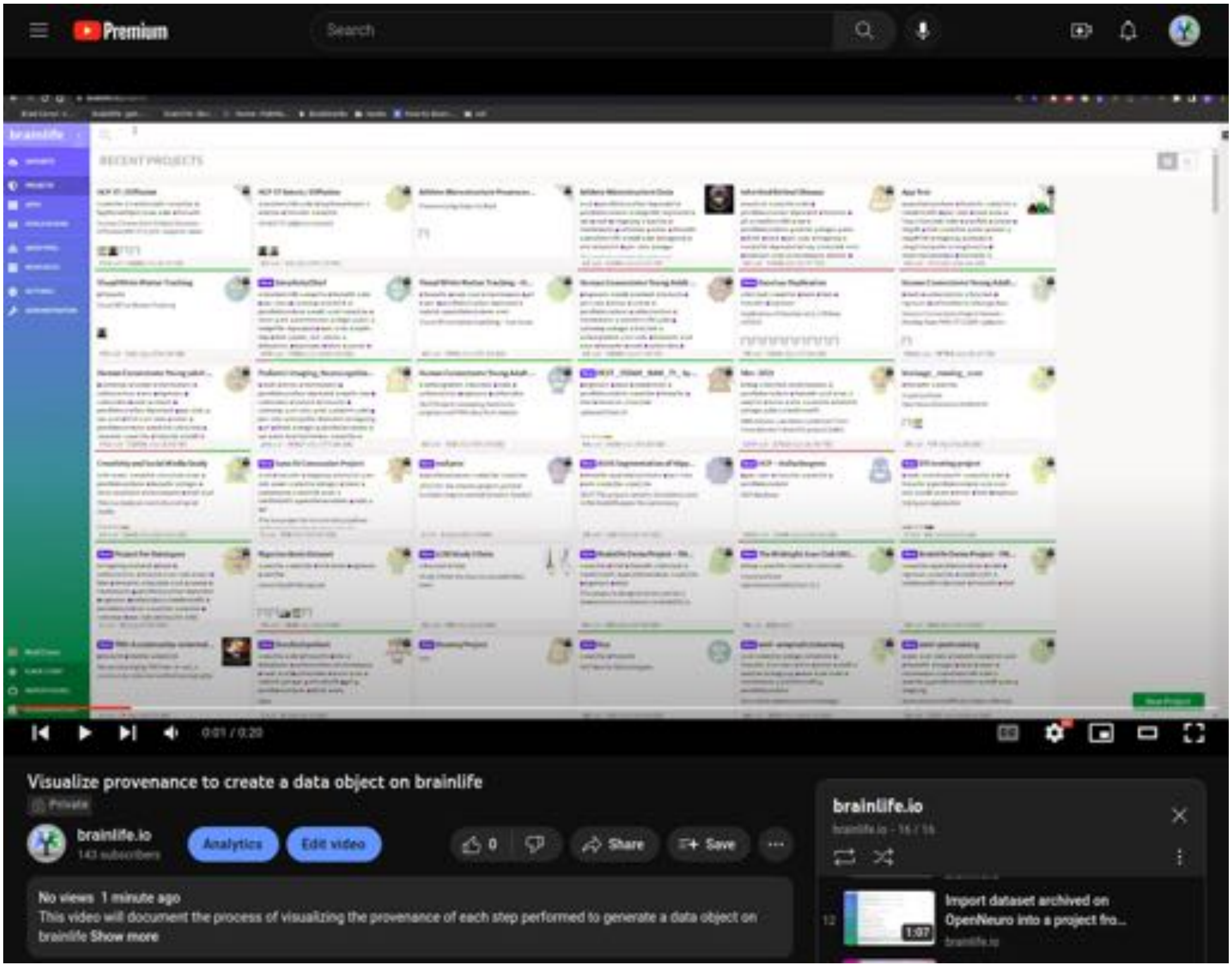

Supplemental Video 10. A video documenting the process of monitoring the steps taken to generate a datatype (provenance). https://youtu.be/NzUObf8_x7g

In addition to the publication of the Notebooks, brainlife.io automatically keeps track of each individual step performed to obtain a specific datatype (i.e. provenance) (**Video S10**). This visualizer contains all of the information a user might need to validate that the proper steps were taken, and for any outsider users or reviewers to rerun their analysis steps for purposes of replication. With this goal in mind, brainlife.io will also generate a script for any data object to reproduce the individual steps to create that object locally (`reproduce.sh`; **Video S11**). Finally, upon completion of processing and analysis, researchers can Publish their datasets, Pipeline rules, and Analysis notebooks directly on the platform via the Publications tab (**Video S12**). All of these individual features are designed with the goal of increasing the reproducibility of processing and analyses performed via the platform.

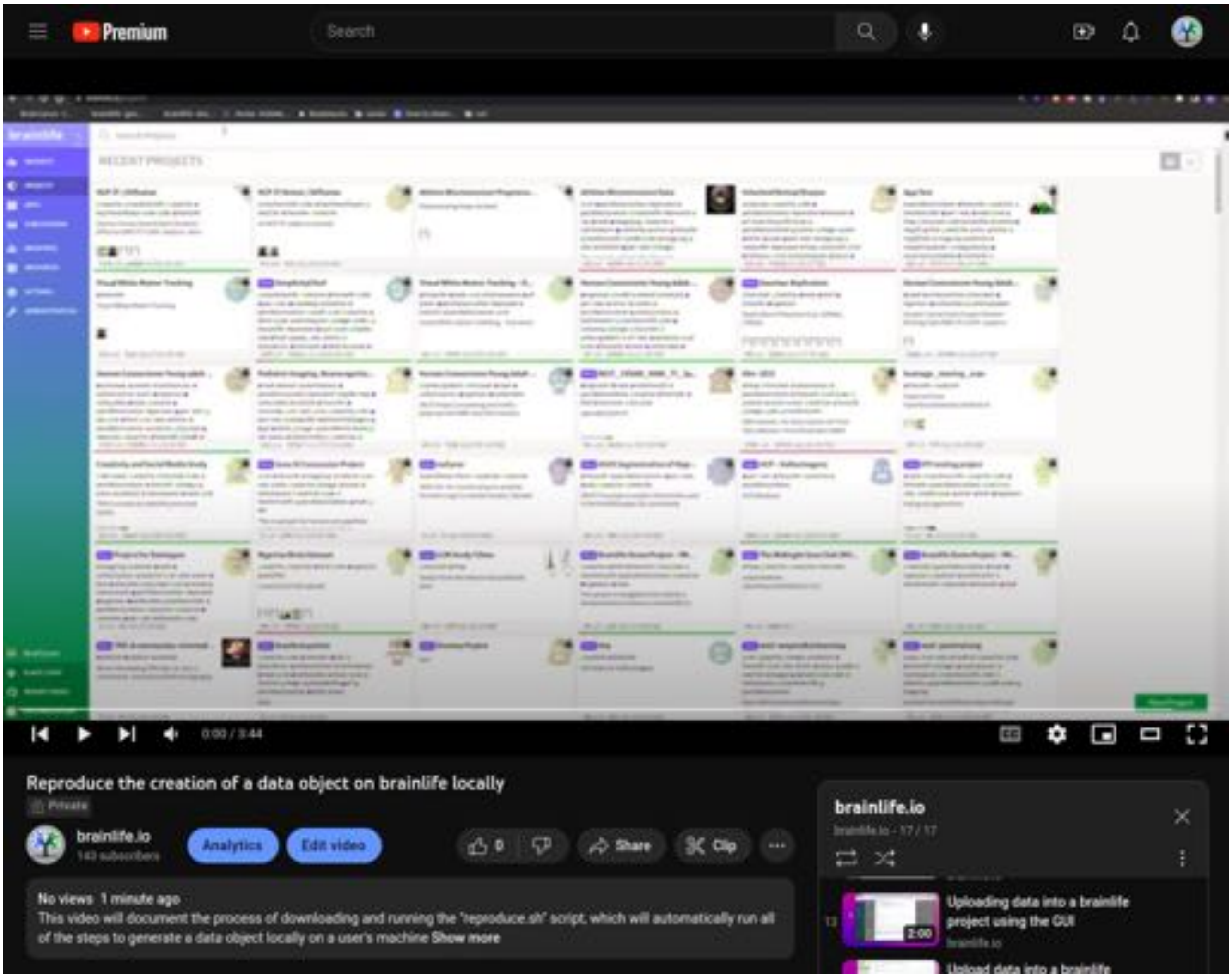

Supplemental Video 11. Video documenting the process of replicating the generation of a data object via a single bash script that can be run on any machine (reproduce.sh). https://youtu.be/YMCFU0aQhvI

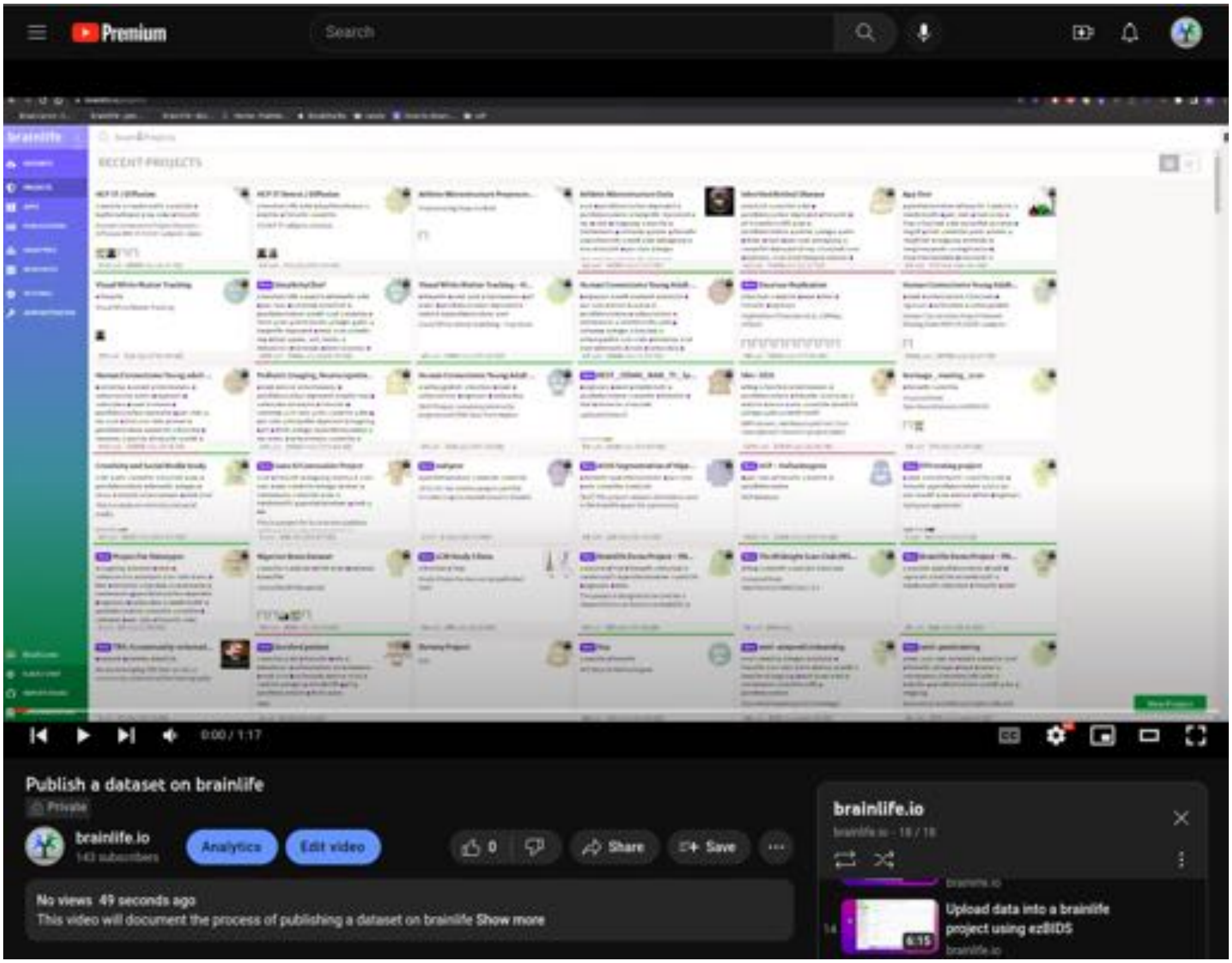

Supplemental Video 12. Video documenting the process of creating a Publication on brainlife.io. https://youtu.be/aUvjuEihWJA

End-to-end reproducible scientific workflow

brainlife.io automatically tracks all actions performed by researchers during data analysis. Data object IDs, Apps versions, and parameter sets used to launch an App, resources used, error logs, etc are all tracked automatically by brainlife.io The full sequence of steps from data import to preprocessing, analysis and publication is captured by the platform and is used to build a record of all the actions a researchers performed while implementing a data analysis study.

A graph describing provenance metadata for each Datatype can be visualized using the provenance visualizer and downloaded (see **Video S10**). Also, a linux shell script is automatically generated to allow the reproduction of full processing sequences (**Video S11**).

Finally, a single record containing data objects, Apps, and Jupyter Notebooks used in a study can be made publicly available outside the platform in a single record addressed by Digital Objects Identifiers (DOI) [51]. Whereas all other existing systems provide users with technology to track analysis steps manually or require the use of coding, *brainlife.io* tracks automatically and does not require coding. This automation technology lowers the barriers of entry to reproducible and transparent large-scale neuroimaging data analysis.

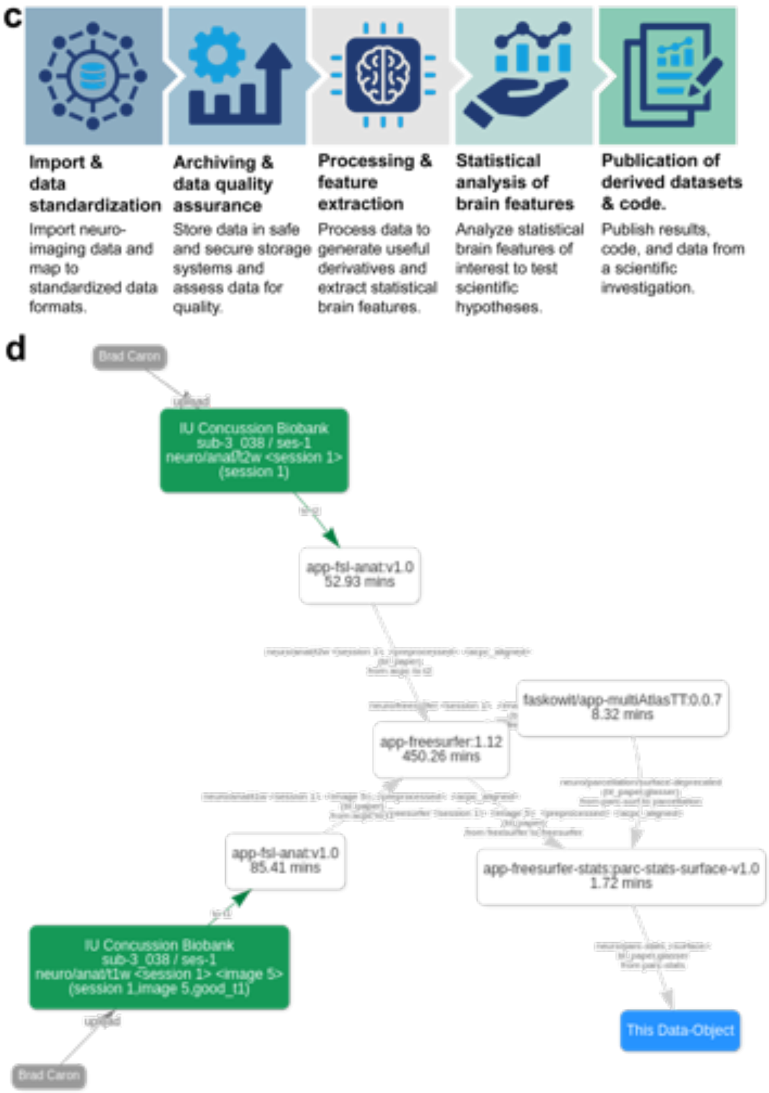

Supplemental Figure 3c,d. End-to-End steps to reproducible computational analysis. a. Pictorial description of the end-to-end workflow for performing a scientific investigation using neuroimaging data. First, data is collected from

measurement systems including MRI and MEG. Following this, data is converted into workable data formats, including NIFTI, .tsv, and .json files, or into standardized file formats including BIDS. Following conversion, data is preprocessed where common artifacts are removed to increase data quality. Model fitting and brain segmentation can then be performed on this cleaned, preprocessed data. Following this, quality assurance (QA) efforts are usually undertaken to ensure the data is of a high enough standard for publication. If the data does not meet a high standard of quality, adjustments to the preprocessing and model fitting steps can be performed (*circular arrows*). Following this, statistical brain features of interest are extracted in order for statistical analyses to be performed. Once the analyses are finalized, researchers then publish their results, data, and code to the greater scientific community. All of these steps are supported by brainlife.io. **b.** Visualization of data "provenance" automatically generated for each archived data object on brainlife.

Neuroimaging investigations involve a common workflow from data collection to study publication (**Fig. S3c**). Data are first either collected from neuroimaging measurement systems, including MRI and MEG scanners. Following collection, data is then converted to standardized file formats before they can be used by the researcher. From here, common artifacts are removed from the data in a series of preprocessing steps. Once the data is cleaned, models can be fit, brain structures can be segmented, and quality assurance assessments are performed. If any mistakes occurred in the previous steps, adjustments can be made to each individual step in order to increase data quality. Only once the data are of high enough quality are statistical brain features of interest extracted, and statistical analyses are performed on the extracted features. Final results, data, and code are then published to the greater scientific community to increase transparency and data gravity of the investigation. Brainlife.io serves each step following data collection, with each step of the workflow tracked in order to increase reproducibility.

Supplemental platform evaluation

Supplemental platform utilization

brainlife.io was developed with a FAIR model and made available worldwide. Any researcher can create an account on *brainlife.io*, although all new accounts are reviewed by the project team. *brainlife.io* first became publicly available in 2018. We tracked the usage of *brainlife.io* in the past 60 months. The platform community, utilization, and research assets have grown steadily since project inception (**Fig. 3** and **Fig. S2c** and **S4**). At the time of writing, over 2,341 users across 43 countries have created a *brainlife.io* account. Over 1,542 active users submitted more than 10 jobs per month (**Fig. 3a**). There were 3,439 data management Projects. The *brainlife.io* developers' community had implemented 530 data processing Apps comprising 2,438,998 lines of code (top 50 apps), and these had been used to process over 270 TBs of data for a total of 3,951,372,037,289 hours of compute time. Apps success rate on average has been 65.4% across 6,710,091 total job submissions (the estimates contain high-failure rate App test-calls). This level of interest and reach, even prior to a formal publication describing the platform, is a testament to *brainlife.io*'s potential for growth and impact.

Researchers ranging from undergraduate students to faculty have already used *brainlife.io* (**Fig. 3b**). The Apps used spanned various aspects of the neuroimaging data lifecycle. The most frequently used Apps pertained to tractography (22%), model fitting (15%), and ROI generation (12%). Community-developed software libraries provided the foundations for data processing Apps, including Nibabel, Freesurfer, FSL, DIPY, MRTrix, Connectome Workbench, and MNE-Python. Terabytes of data have been uploaded (72%) or imported from OpenNeuro.org (22%), the Nathan-Kline Institute data sharing projects (3%; [44,46,52]), and other sources. Early community attention and adoption preceded this publication describing the project and platform. The worldwide platform access highlights the global need for technology like *brainlife.io* (**Fig. S2e**).

Apps performance evaluation

Brainlife.io, like any technology, is not *failure-proof*. To examine the rate at which brainlife.io Apps fail, we collected data regarding the failure rates of all Apps across the platform. Since the beginning of the platform, jobs processed on brainlife.io have had a 34.6% failure rate across 6,710,091 submissions, with half estimated to be due to initial App testing and development (**Fig. S3e**).

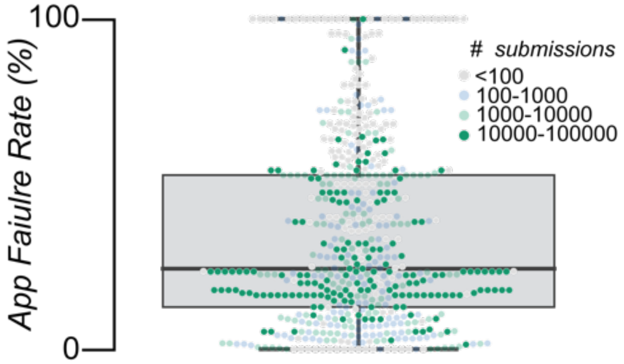

Supplemental Figure 3e. Brainlife.io processing is not error-proof. Distribution of brainlife.io App failure rates (percentage) across all 568 Apps and their respective submissions. Box-and-whisker plot indicates the overall average failure rate across all Apps (*dark black line*), 25th and 75th percentiles (*box*), and overall range (*whiskers*). Each dot is an individual App's failure rate. Colors represent the number of submissions for each App (*grey*: 0-100 submissions, *light blue*: 100-1,000 submissions, *light green*: 1,000-10,000 submissions, *dark green*: 10,000-100,000 submissions).

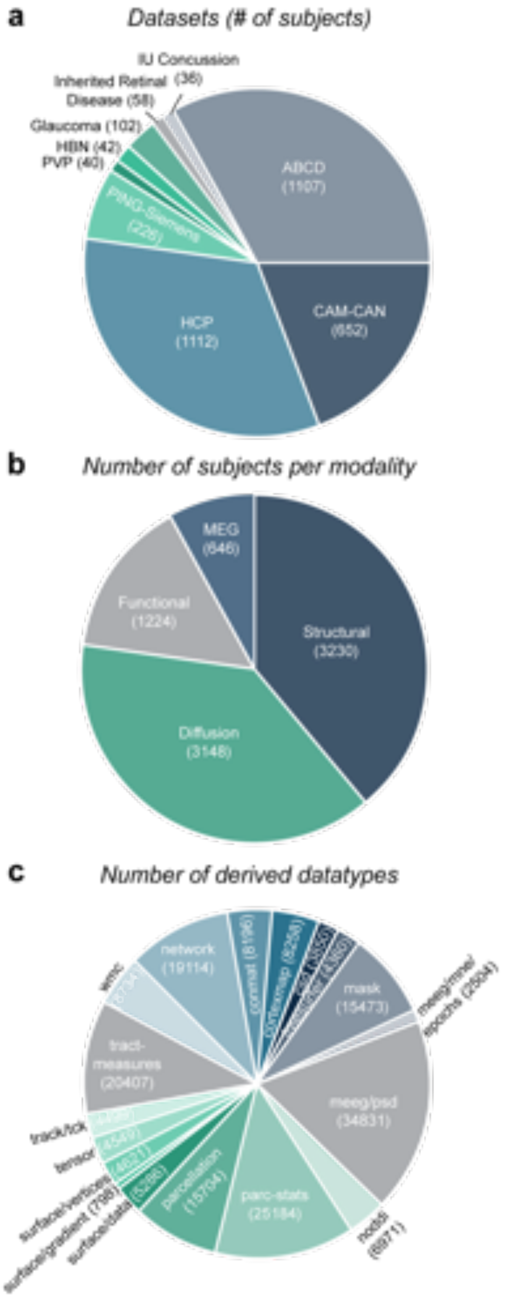

Supplemental Figure 4a-c. Overview of data used for the study. a. Number of subjects across all datasets examined. **b.** The number of subjects per imaging modality. **c.** The number of brainlife.io datatypes (i.e. freesurfer, parc-stats, tractmeasures, track/tck, NODDI, tensor, csd, mask, network, conmat, parcellation, cortex map, wmc, meeg/psd, meeg/mne/epochs, surface/data, surface/vertices, surface/gradient) derived across all subjects and datasets examined.

Supplemental platform testing

The effectiveness of the technology to provide good quality results were evaluated. We performed system load experiments by processing large amounts of data and evaluating the results obtained. These experiments were performed to demonstrate the ability of the platform to serve accurate data processing and analysis at scale.

Our experiments focused on the four axes of scientific transparency,[53] namely: data processing validity [54] (**Fig. 4a-e;** Fig. S4d-g), reliability (**Fig. 4f-j;** Fig. S4h-i), reproducibility (Fig. S4j-n), and replicability (**Fig. 7;** Fig. S7a,b). Four data modalities (sMRI, fMRI, dMRI, MEG) were evaluated using the test-retest HCP_{TR} [55], the Cam-CAN [56], the HBN [52], and the ABCD[57] dataset. For all experiments, the Pearson's correlation (*r*) and root mean-square-error (*rmse*) were computed for each comparison using data products derived from apps on brainlife.io, where high

correlations and low *rmse* would provide strength of evidence in each experiment. Herein we describe the definitions of success for each experiment and the methods used to assess the performance of the platform. For all reported correlations and root mean-square-error values for the data validity and reliability experiments, see **Table S3**.

A total of 12 different datasets comprising over 4,200 participants were processed (**Fig. S4a**), of which 3,200 participants had sMRI, 3,100 had dMRI, 1,200 had fMRI, and 650 had MEG data objects (**Fig. S4b**). Derived data products included cortical parcel volumes, white matter profilometry, functional and structural networks properties, functional gradients, and peak alpha frequency (**Fig. 4**, **Fig. S4c**). In sum, over 193,000 objects and 22 Terabytes of data were generated for the experiments, using over 30 Apps (**Table S3**).

For each of the four data modalities, data processing validity was defined as the ability of a processing step to accurately reflect ground-truth properties of the brain. Data processing validity was estimated by comparing values obtained using *brainlife.io* Apps (see **Table S3**) against data preprocessed by the data originator (HCP or Cam-CAN depending on the data modality). Cortical parcel volumes were estimated from minimally processed HCP_{TR} data using *brainlife.io* Apps A0, A462, A23, A272, and A464. Volume estimates were compared to corresponding estimates provided by the HCP consortium (**Fig. 4a**; $r_{validity}$=0.98, $rmse_{validity}$=570.54mm^3).

Fractional anisotropy (FA) in 61 white matter tracts was estimated using the raw and minimally preprocessed HCP_{TR} dMRI data. The composable processing pipeline comprised of the sequence of Apps: A68, A238, A297, A305, A188, A195, and A361. These Apps were used to process either type of data, with the exception of A68,[30] for which only raw data was used. The average FA for each tract was compared between these two processing methods (**Fig. 4b**; $r_{validity}$=0.95, $rmse_{validity}$=0.018).

Functional connectivity estimates between 117^2 nodes-pairs [58] were estimated using the raw and minimally preprocessed HCP_{TR} fMRI data. A160, A23, A369, and A532 were used to process either dataset, with the exception of A160, [22] which was only used with raw data (**Fig. 4c**; $r_{validity}$=0.89, $rmse_{validity}$=0.12).

In addition, functional gradients [59,60] were computed on 400 nodes estimated on raw and minimally processed HCP_{TR} fMRI data using A604 and A574. The average primary gradient within each node was compared between raw and minimally processed data (**Fig. 4d**; $r_{validity}$=0.59, $rmse_{validity}$=0.036).

Finally, the peak alpha frequency (Hz) was estimated from the Cam-CAN MEG data filtered by *brainlife.io* apps and data Maxwell-filtered by Cam-CAN using A476 and A531 [61,62]. Peak alpha values were compared between the two differently processed datasets (**Fig. 4e**; $r_{validity}$=0.94, $rmse_{validity}$=0.30 Hz).

For each data modality, data preprocessing reliability was defined as the ability to produce the same results given repeated *acquisitions* from within a participant. Data processing reliability was examined by comparing brain features estimated using brainlife.io Apps pipelines using either test-retest HCP_{TR} data or a median split of Cam-CAN MEG data.

Cortical parcel volumes from the test and retest dataset of HCP_{TR} were obtained using A23, A272, and A464 brainlife.io Apps (see **Table S3**) and compared (**Fig. 4f**; $r_{reliability}$=0.99, $rmse_{reliability}$=278.11mm^3).

Mean FA from 61 white matter tracts was estimated independently for *test* and *retest* HCP_{TR} dMRI data using A238, A297, A305, A188, A195, and A361. The average FA for each tract was compared between test and retest conditions ($r_{reliability}$=0.93, $rmse_{reliability}$=0.017) (**Fig. 4g**).

Functional connectivity estimates between 117^2 nodes-pairs were estimated using the test and retest HCP_{TR} fMRI data using A23, A369, and A532 (**Fig. 4h**; $r_{reliability}$=0.73, $rmse_{reliability}$=0.19).

In addition, functional gradients were computed on 400 nodes estimated on test and retest HCP_{TR} fMRI data using A604 and A574. The average primary gradient within each node was compared between datasets (**Fig. 4i**; $r_{reliability}$=0.85, $rmse_{reliability}$=0.026).

Finally, the frequency of the amplitude peak (between 8 and 13 Hz from the occipital magnetometers and gradiometers) was estimated from two median splits of Maxwell-filtered Cam-CAN MEG data using A529 and A531. Peak alpha frequency values were compared between the two datasets ($r_{reliability}$=0.85, $rmse_{reliability}$=0.48 Hz; **Fig. 4j**). All estimated validity and reliability estimates are reported in **Table S4**.

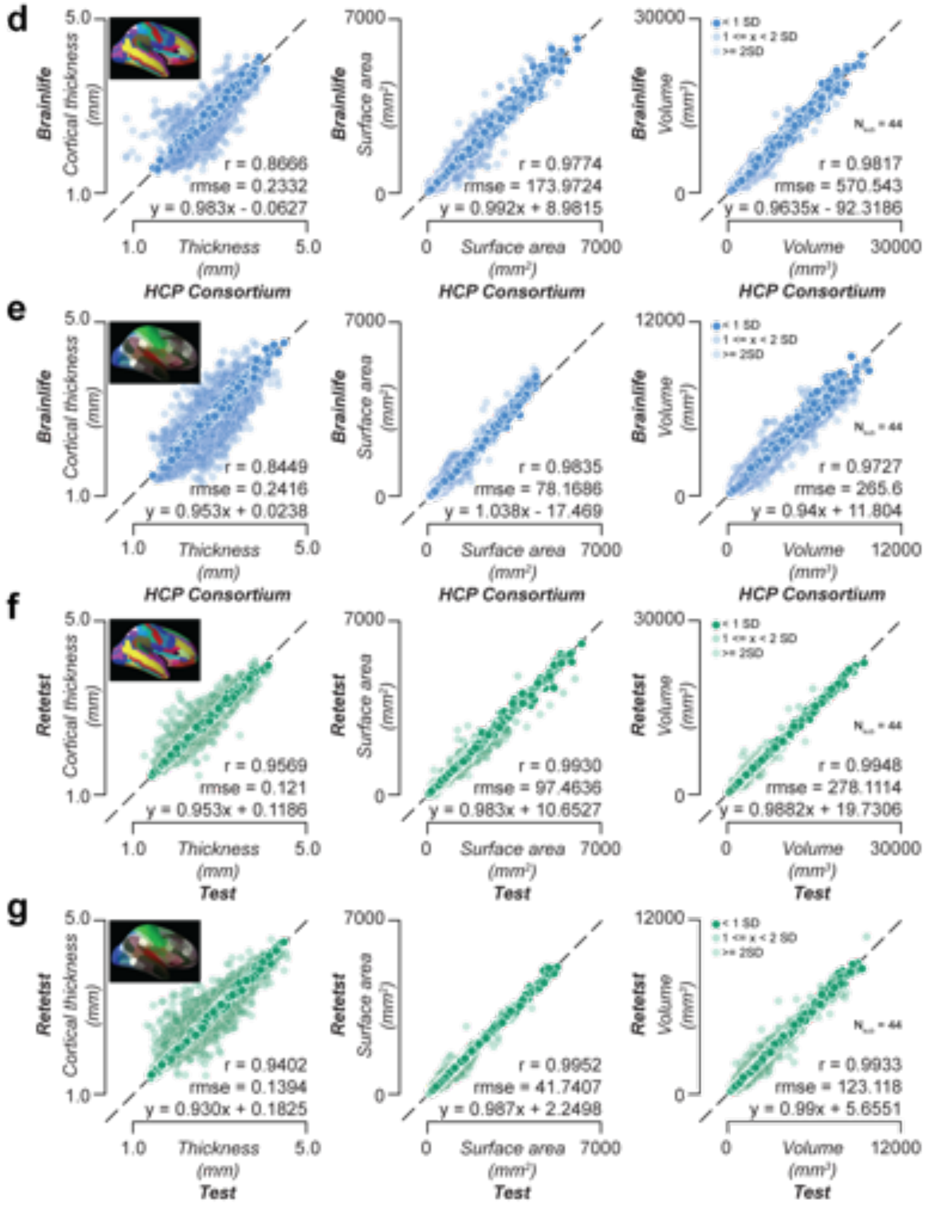

Supplemental Figure 4d-g. Processing with brainlife.io is valid and test-retest reliability is high. ***Top rows:*** Validity measures derived using the HCP_{TR} data preprocessed and provided by the HCP Consortium compared to data preprocessed on brainlife.io. Each dot corresponds to the ratio for a given subject between data preprocessed and provided by the HCP Consortium vs data preprocessed on brainlife.io in a given measure for a given structure. Pearson's correlation (*r*), root mean squared error (*rmse*), and a linear fit between the test and retest results were calculated and provided. **a.** Destrieux Parcel thickness (mm), surface area (mm^2), and volume (mm^3). **b.** HPC-mmp Parcel thickness (mm), surface area (mm^2), and volume (mm^3). Dark colors represent data within +/- 1 standard deviation. 50% opacity represents data within 1-2 standard deviations. 25% opacity represents data outside 2 standard deviations. ***Bottom rows:*** Test-retest reliability measures derived from derivatives of the HCP_{TR} dataset generated using brainlife.io. Each dot corresponds to the ratio between a test-retest subject and a given measure for a given structure. Pearson's correlation (*r*), root mean squared error (*rmse*), and a linear fit between the test and retest results were calculated and provided. **c.** Destrieux Parcel thickness (mm), surface area (mm^2), and volume (mm^3). **d.** HPC-mmp Parcel thickness (mm), surface area (mm^2), and volume (mm^3). Dark colors represent data within +/- 1 standard deviation. 50% opacity represents data within 1-2 standard deviations. 25% opacity represents data outside 2 standard deviations.

Computational reproducibility was defined as the ability to produce the same results given repeated *runs* of a processing app. Computational reproducibility was estimated by comparing values obtained from repeated runs of *brainlife.io* Apps.

Cortical parcel volumes were estimated twice from the minimally processed HCP_{TR} data (N_{sub} = 44) using A272. Volume estimates between the repeat run were compared (**Fig. S4j;** $r_{reproducibility}$ = 0.99, $rmse_{reproducilibity}$ = 34.22 mm^3).

Fractional anisotropy (FA) in 61 white matter tracts was estimated from the minimally processed HCP_{TR} data (N_{sub} = 43) using A361. The average FA for each tract was compared between repeated runs (**Fig. S4k**; $r_{reprodubility}$ = 0.99, $rmse_{reprodubility}$ = 0.011).

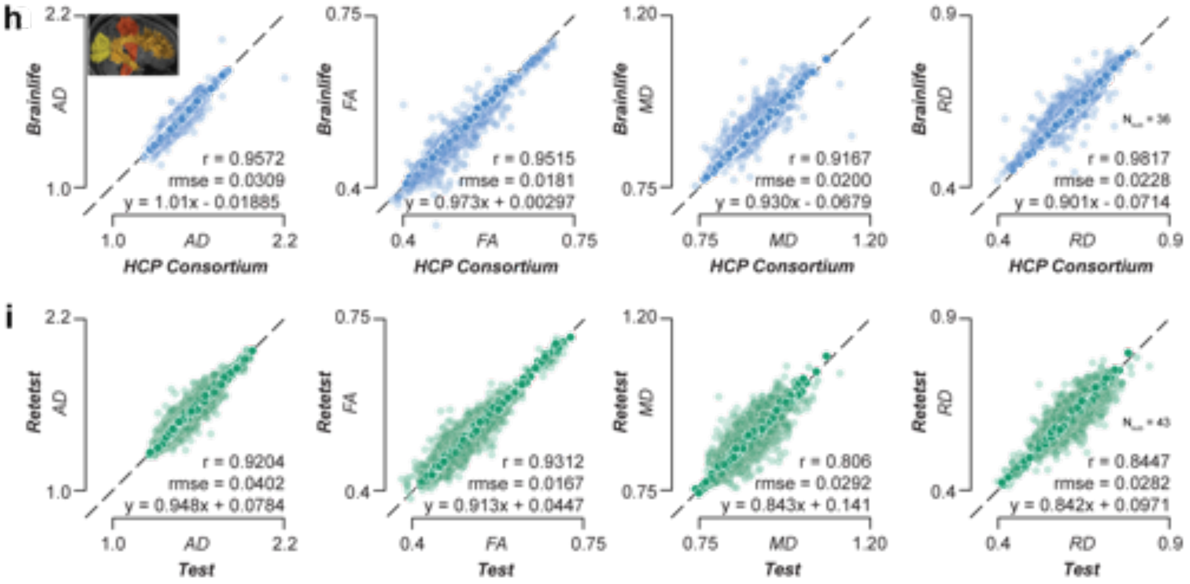

Supplemental Figure 4h-i. Processing with brainlife.io is valid and test-retest reliability. ***Top row:*** Validity measures derived using the HCP_{TR} data preprocessed and provided by the HCP Consortium compared to data preprocessed on brainlife.io. Each dot corresponds to the ratio for a given subject between data preprocessed and provided by the HCP Consortium vs data preprocessed on brainlife.io in a given measure for a given structure. Pearson's correlation (*r*), root mean squared error (*rmse*), and a linear fit between the test and retest results were calculated and provided. **e.** Tract average AD, FA, MD, and RD. Dark colors represent data within +/- 1 standard deviation. 50% opacity represents data within 1-2 standard deviations. 25% opacity represents data outside 2 standard deviations. ***Bottom row:*** Test-retest reliability measures derived from derivatives of the HCP_{TR} dataset generated using brainlife.io. Each dot corresponds to the ratio between a test-retest subject and a given measure for a given structure. Pearson's correlation (*r*), root mean squared error (*rmse*), and a linear fit between the test and retest results were calculated and provided. **f.** Tract average AD, FA, MD, and RD. Dark colors represent data within +/- 1 standard deviation. 50% opacity represents data within 1-2 standard deviations. 25% opacity represents data outside 2 standard deviations.

Functional connectivity estimates between 117^2 node pairs were estimated using the minimally processed test HCP_{TR} data (N_{sub} = 32) using A532. Average node connectivity was compared between repeated runs (**Fig. S4l**; $r_{reproducibility}$ = 1.0, $rmse_{reproducibility}$ = 0.0). In addition, functional gradients were computed on 400 nodes estimated from the Cam-CAN data (N_{sub} = 613) using A574.

Finally, primary gradient values were compared between repeated runs (**Fig. S4m**; $r_{reproducibility}$ = 0.99, $rmse_{reproducibilty}$ = 0.03). Finally, the peak alpha frequency (Hz) was estimated from the Maxwell-filtered MEEG Cam-CAN data (N_{sub} = 501) using A531. Peak alpha values were compared between repeated runs (**Fig. S4n**; $r_{reproducibility}$ = 0.99, $rmse_{reproducibility}$ = 0.0002).

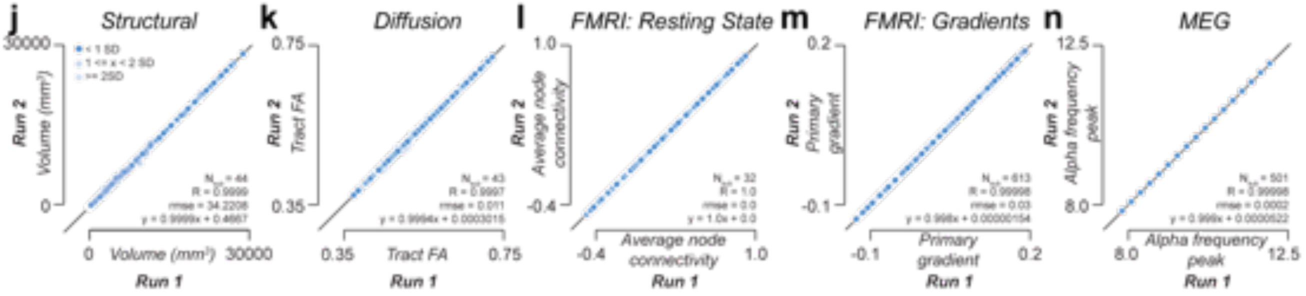

Supplemental Figure 4j-n. **App computational reproducibility.** Computational reproducibility values derived by repeating runs of brainlife.io Apps using the HCP$_{TR}$.dataset and the CAN dataset. Each dot corresponds to the ratio for a given subject between repeated runs of each App for a given structure. Pearson's correlation (*r*), root mean squared error (*rmse*), and a linear fit between the repeated runs was calculated. **a**. Destrieux Atlas Parcels volume (mm^3). **b.** Tract-average fractional anisotropy (FA). **c.** Node-average functional connectivity (FC). **d.** Primary gradient values derived from resting state fMRI. **e.** Peak alpha frequency (Hz) in the alpha band derived from MEG.

Supplemental platform utility for scientific applications

Evaluation of the scientific utility of the platform was performed on over 2,000 participants across three large datasets with participant ages spanning over 7 decades—PING (Pediatric Imaging, Neurocognition, Genetics), HCP$_{s1200}$, (Human Connectome Project Young Adult 1,200) and Cam-CAN (Cambridge Center for Ageing Neuroscience). Multiple brain features were derived, including fractional anisotropy of cortical parcels and within-network functional connectivity of individual Yeo17 networks. Specifically, for structural MRI data, the volumes of the cortical and subcortical structures segmented for each participant were compared to their age at the time of scan acquisition on a per-structure basis. Volume measures were estimated using A464, A462, A272, and A379. For diffusion MRI data, the average FA for each of the white matter tracts segmented for each participant was compared to the participant age at scan acquisition on a per-structure basis. Tract average FA values were estimated using A361. In addition to white matter tract FA, average FA within cortical regions was computed using A383. For resting-state functional MRI connectivity, the average within-network connectivity values, defined as the average connectivity values between all of the nodes within each resting state network of the Yeo17 parcellation, was compared to the participant's age at scan acquisition. Network connectivity matrices were estimated using A532. For resting-state functional gradients, the cosine distance of the primary gradient for each of the resting state networks in the Schaffer parcellation was compared to the participant's age at scan acquisition. Gradients were mapped using A574. Finally, for MEG data, the peak frequency in the alpha band across all nodes was compared to the participant's age at the time of acquisition. Peak frequency was estimated using A531. For structural and diffusion MRI data, data from all three data sources (HCP$_{s1200}$, Cam-CAN, PING) was used. For the functional MRI data, data from only the HCP$_{s1200}$ and Cam-CAN data sources were used. For the MEG data, only the data from the Cam-CAN data source was used. To assess the relationship between each of the measures and age within each structure investigated, a quadratic model ($y_{feature} = ax^2_{age} + bx_{age} + c$) was fit across all of the data, and a linear regression was fit within each data source, using functions from scikit-learn[63] Two additional examples are presented in **Fig. S5**, specifically the average fractional anisotropy (FA) or cortical V1 (**Fig. S5a**) and the within-network average functional connectivity within the default mode (A) network derived from the Yeo17 atlas (**Fig. S5b**). The quadratic model (R^2=0.12 ± 0.015 s.d.) for these two examples demonstrated the expected inverted U-shape trajectory, with the mean quadratic term (*a*) across each data modality being negative (-3.70x10^{-6} ± 6.60x10^{-6} s.d.).

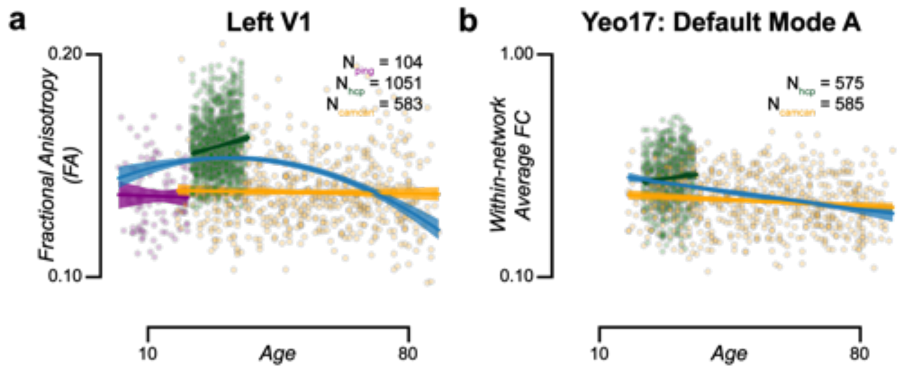

***Supplemental Figure 5*. Additional examples of inverted U-shaped trajectories.** Relationship between age of subject and **a.** Cortical fractional anisotropy (FA) of the left V1, **b.** Within-network average functional connectivity (FC) from the Yeo17 Default Mode - A network. These analyses include subjects from the PING (*purple*), HCP_{s1200} (*green*), and CAN (*yellow*) datasets. Linear regressions were fit to each dataset, and a quadratic regression was fit to the entire dataset (blue).

Supplemental replication and generalization

In addition to the replication experiments, five sets of generalization experiments were performed (**Fig. 6; Fig. S6a,b**). First, we tested *brainlife.io*'s ability to replicate scientific results from five previous studies [64–66]. A key finding from each previous study was identified as the target found to be reproduced. We then followed the processing methods as outlined in the original study but performed these processing methods using *brainlife.io* Apps. Post-processing analyses were performed in line with the original study using *brainlife.io*-hosted Jupyter Notebooks (see **Table S2**). Replicability success was measured by comparing trends in the data obtained with brainlife.io Apps and those reported in the original study.

Replicability was defined as the ability to reproduce individual experiments already published by other members of the scientific community. Within replicability are two pillars: the ability to reproduce results within the *same* dataset, and the ability to generalize results to *new* datasets. Three sets of experiments were performed to assess the ability of the platform to replicate previously published findings. The first experiment attempted to replicate a reported negative correlation between a cortical region's thickness and its tissue orientation organization within the HCP_{s1200} dataset. Cortical regions found within the HCP multi-modal parcellation (hcp-mmp) parcellation were first mapped to each participant's Freesurfer surfaces using A23. Brainlife apps A464, A462, A272, and A379 were then used to map and estimate each region's cortical thickness and orientation dispersion index (ODI), respectively. The relationship between ODI and cortical thickness was assessed by computing the correlation between these values across all parcels within the hcp-mmp parcellation (**Fig. 6a**). The second experiment attempted to replicate the improved ability to segment the Inferior Longitudinal Fasciculus from the HCP_{s1200} dataset (**Fig. S6a)** [32]. The Right Inferior Longitudinal Fasciculus (ILF) was segmented from the HCP_{s1200} dataset using an automated segmentation algorithm (A174). The same improved ability of tract segmentation was obtained (**Fig. S6a**; AUC_{LAP} = 0.77, $AUC_{NN\_DR\_MAM}$ = 0.66). The third study used to assess replicability investigated the performance of an automated hippocampal subfield segmentation as compared to hand-drawn regions of interest (ROIs)[67]. The original implementation was performed with a dice coefficient ranging from 0.525-0.823. An App (A262) was created to implement this segmentation on brainlife. The method was implemented on participants from the UPENN-PMC dataset. Improved model performance was obtained for segmenting hippocampal subfields (**Fig. S6b**; dice range = 0.838-0.945).

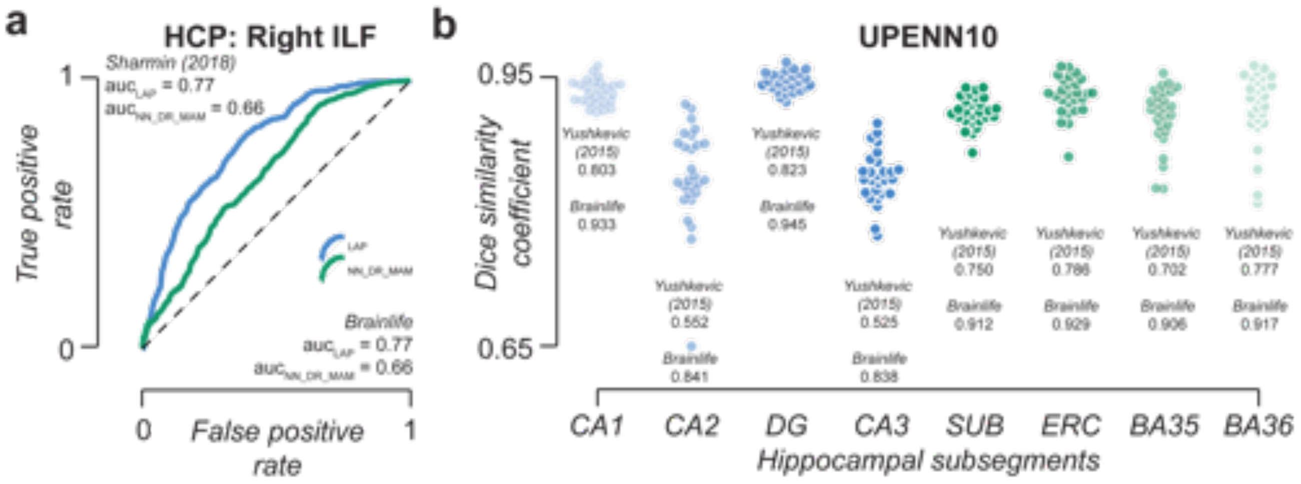

Supplemental Figure 6a,b. **Replication of previous studies using brainlife.io a.** Receiver operator curves (ROC) comparing the performance of segmentation of the Right ILF using two automated segmentation methods (LAP: blue, NN_DR_MAM: green) in a subset of the HCP $_{S1200}$ dataset (N_{sub} = 15). **b.** Dice coefficients between manual and automated segmentation of the hippocampus using AHSS method in UPENN dataset.

In addition to the replication experiments, three sets of generalization experiments were performed. The first experiment attempted to generalize the same relationship between a cortical region's thickness and orientation dispersion index found within the HCP_{s1200} dataset to the Cam-CAN dataset (**Fig. 6a**). *brainlife.io* Apps A464, A462, A272, and A379 were then used to map and estimate each region's cortical thickness and orientation dispersion index (*ODI*), respectively. The relationship between ODI and cortical thickness was assessed by computing the correlation between these values across all parcels within the hcp-mmp parcellation. A negative trend of about half the magnitude of the original was estimated (**Fig. 6a**; $r_{Cam\text{-}CAN\text{-}brainlife}$ = -0.28 vs. $r_{original}$). The second and third experiments attempted to generalize a relationship between the average quantitative anisotropy (QA) and fractional anisotropy (FA) of the left and right uncinate with the presence of stressful life events as an adolescent (**Fig. 6b,c**). The second experiment assessed tract organization within the UF of 42 participants from within the HBN dataset using A423 to extract the UFs and to map QA to each, respectively. These values were then compared to the number of negative life events as reported on the Negative Life Events Schedule (NLES) collected by the HBN group. A negative relationship between UF QA and number of stressful life events was identified (**Fig. 6b** r_{HBN_LEFT} = -0.35, p-value < 0.05; r_{HBN_RIGHT} = -0.39, p-value < 0.05). The third experiment attempted to find the same relationship using FA within 1,107 participants from the ABCD dataset. For this, an end-to-end white matter processing pipeline composed of A68, A238, A297, A305, A188, A195, and A361 was used to extract the UF and to map FA to each tract. These values were then compared to the measure of early life stress was estimated as a composite score by z-scoring separately and then summing across the following questionnaires: traumatic life events reported by the parent, environmental and neighborhood safety reported by both parent and adolescent, and the Family Environment Scale-Family Conflict Subscale Modified from PhenX reported by both parent and adolescent [68]. A negative relationship between UF FA and the composite score was estimated in the left- and right-UF (**Fig. 6c** r_{ABCD_LEFT} = -0.12, p-value < 0.001; r_{ABCD_RIGHT} = -0.09, p < 0.01).

Supplemental to detecting disease

The final two tests to demonstrate the platform's potential and scientific utility focused on identifying human disease biomarkers. We examined data from multiple clinical populations including sports-related concussions, glaucoma, Stargardt's, Choroiderema, and healthy populations who have experienced stressful life events to assess the ability to identify unique clinical characteristics using the platform; **Fig. 7**). It has been reported that concussions can alter brain tissue properties both in the cortex and in deep white matter tracts [69]. Here, the differences in cortical white matter tissue in concussed and matched controls were tested. Specifically, for sports-related concussion, 10 concussed athletes and 10 healthy within-sport control athletes from the Indiana University Acute Concussion dataset (in prep) was used. FA was estimated in 358 cortical parcels from the Human Connectome Project multimodal parcellation [70] using a pipeline composed of A23, A272, A379, and A464. The distribution of FA in the superior temporal sulcus (STS) is reported in **Fig. 7a**. One example athlete with strong

post-concussive symptoms and low FA (red arrow) is compared to the distribution of controls (gray) to demonstrate the ability to detect meaningful changes in brain tissue following a concussive event.

Changes in the visual white matter as a result of eye disease have been reported [38,71–74]. Individuals with Stargardt's disease (a deterioration of the human retina initiating in the central fovea), and Choroideremia (retinal deterioration initiating in the visual periphery), were compared to healthy controls. Optical coherence tomography (OCT) data were processed using A346 (**Fig. 7b**). Photoreceptor complex thickness (microns) was estimated for foveal and peripheral (0-1 and 7-90 degrees of visual eccentricity) regions. Choroideremia patients showed similar levels of photoreceptor complex thickness compared to healthy controls in the foveal bundle but deviated in the peripheral bundle (**Fig. 7b**). This trend was the opposite for Stargardt's participants. To study the degree to which retinal damage affects the brain's white matter (the optic radiation, OR), data were processed using a series of Apps (A273, A462, A187, A414, A233, A361, A68, A238, and A346). Visual eccentricity maps in area V1 were separated between foveal (0-1° of visual angle) and peripheral (7-90° of visual angle) regions [75,76]. Tractography was used to separate OR bundles projecting to the foveal and peripheral maps, and average FA profiles for each group and bundle were computed [77] [78,79]. Results show a reduction in FA in the component of the OR projecting to foveal (but not peripheral) V1 in Stargardt's patients (**Fig. 7b**, blue). Results also show a reduction in FA in the Choroideremia patients' peripheral (but not foveal) bundle (**Fig. 7b**, blue). Taken together, these results demonstrate the ability of the technology implemented in the platform to measure disease biomarkers.

Supplement to quality control at scale

To assure quality in processed data, brainlife.io provides a unique approach to quality assurance (QA). State-of-the-art approaches to QA provide users with the ability to assess quality after data processing is compiled into QA reports [22,23,80–83]. The platform supports QA reports outputted by state-of-the-art processing pipelines (A160, A246, A160, A462, A423, A399), as well as via QA images, which can be assessed by individuals or groups. Here we propose an additional approach to QA via *normalized reference ranges*, in which brain properties derived from many participants, modalities, and sources of variability are collated together for quick identification of aberrant brain derivatives [84].

Normalized reference ranges were generated and are served on the brainlife.io platform in addition to the standard QA reports that are generated within Apps for individual datasets. To generate the reference ranges, the brain properties derived from the three datasets (PING, HCP_{s1200}, and Cam-CAN) and four data modalities in 1,751 participants generated for the load testing of the platform (as described in the previous sections) were curated (removed of outliers) and collated for *brainlife.io* datatype. For each datatype, a single JSON file was created reporting the mean and ±1 and 2 standard deviations of the outlier-removed measure (e.g., the volume of a brain parcel, fractional anisotropy of a white matter tract, functional connectivity of a network, power-spectrum density across MEG sensors, etc). The JSON files were saved on a repository (github.com/brainlife/reference) and the brainlife.io datatype validator service made use of the JSON to automatically visualize a plot of the data. We call these JSON files reference datasets. Users utilizing Apps (A272, A463, A483, A361, A530, A531, A532) that generate datatypes for which a reference dataset was created will find the values of the features estimated by the App on any new dataset overlaid on top of the corresponding reference dataset (see **Fig. S8**). We report examples of reference dataset plots for four major datatypes, with outliers data overlaid on top (**Fig. 8a-d**) and the final *reference* datasets for each datatype and data source.

A critical aspect to democratizing big data neuroscience is the ability of investigators to perform quality assurance (QA), because there is no value in increasing dataset size unless quality can be assured for each dataset. State-of-the-art approaches provide users with the ability to assess quality after data processing is compiled into QA reports [22,23,80–83], or through the use of citizen science [85]. The brainlife.io platform supports visualization of the QA reports outputted by state-of-the-art processing pipelines (A160, A246, A160, A462, A423, A399), as well as via QA images, which can be assessed by individuals or groups. Here we propose an additional approach to QA via *normalized reference ranges*, in which brain properties derived from many participants, modalities, and sources of variability are collated together for quick identification of abnormal brain derivatives [84]. For more details of the generation of these reference ranges, see **Methods.** Here we provide an example of the brainlife.io visualizations of reference datasets (**Fig. S8**).

The brain properties derived from the three datasets (PING, HCP_{s1200}, and CAN) and four data modalities in 1,751 participants generated for the load testing of the platform (as described in the previous sections) were curated (removed of outliers) and collated for brainlife.io datatype. For each datatype, a single JSON file was created reporting the mean and ±1 and 2 standard deviations of the outlier-removed measure (e.g., the volume of a brain

parcel, fractional anisotropy of a white matter tract, functional connectivity of a network, power-spectrum density across MEG sensors, etc). The JSON files were saved on a repository (https://github.com/brainlife/reference) and the brainlife.io datatype validator service made use of the JSON to automatically visualize a plot of the data. We call these JSON files reference datasets. Users utilizing Apps (A272, A463, A483, A361, A530, A531, A532) that generate datatypes for which a reference dataset was created will find the values of the features estimated by the App on any new dataset overlaid on top of the corresponding reference dataset (**Fig. S8**).

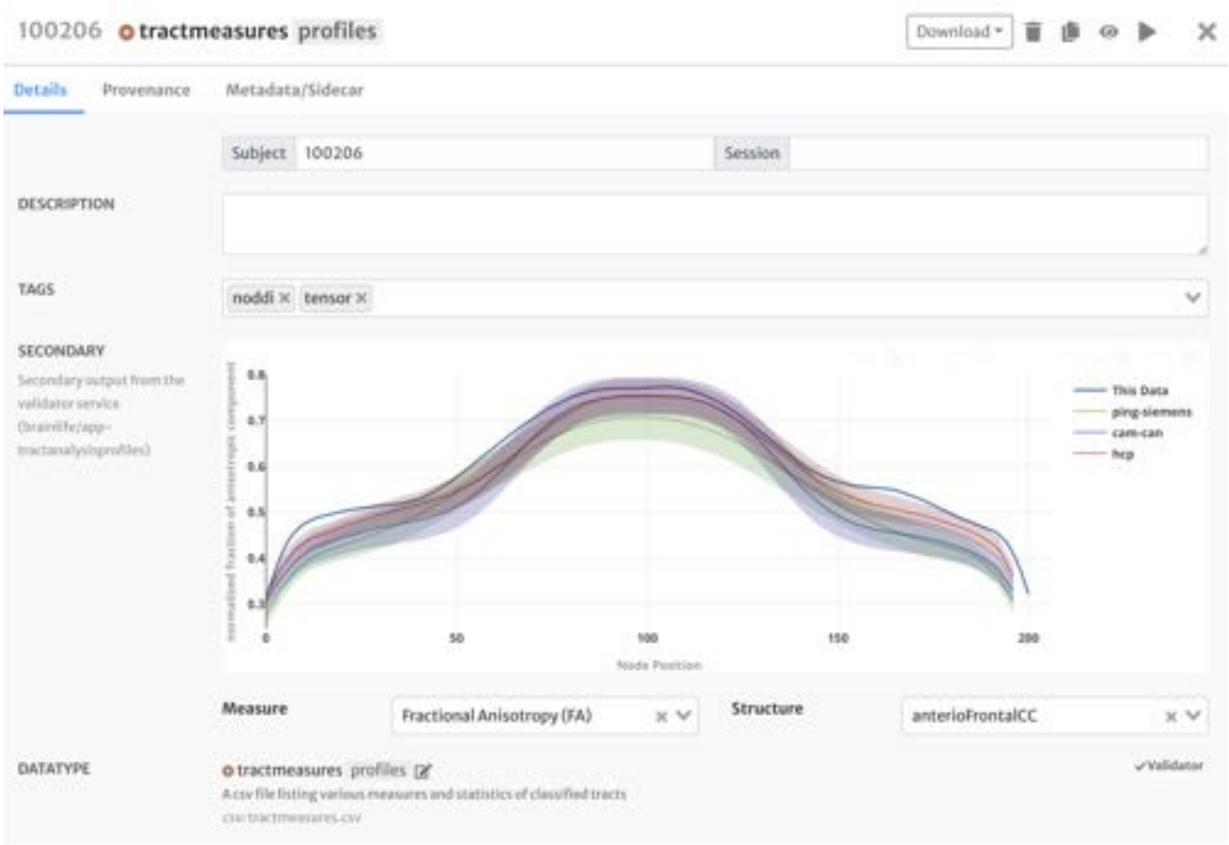

Supplemental Figure 8. **brainlife.io interface can visualize reference datasets.** Validation services for datatypes containing statistical feature information automatically generate a visualization of newly generated data (*blue line*) overlaid on reference dataset ranges for the three data sources used to generate reference datasets (i.e. HCP_{S1200} (*red*), PING (*green*), CAN (*purple*). These reference ranges can be used to quickly assess the quality of the estimated statistical features of interest.

Public services for promoting transparency and data gravity in neuroscience research.

In the previous section, we described the system architecture for the platform. These components and architectures were implemented in order to reduce barriers of entry to performing neuroimaging investigations and to ultimately increase data gravity and representation in neuroscience. These goals coincide with a push within the neuroimaging community to increase data gravity and representation by providing standardization of data formatting, software libraries, and computing resources. From this push has come an ever-growing list of publicly available services and platforms for increasing data gravity in neuroimaging. However, there currently exists only one compiled list of the services available [86]. To address this, and to help increase transparency in neuroscientific research, we provide a non-comprehensive list of currently available services and platforms for increasing data gravity across the greater neuroimaging community (**Table S5**). This list is not designed to cover all currently available services and platforms, but to provide a sense of the scope of available technologies developed by the neuroscientific community.

The FAIR principles.

Recently, it has been proposed that platforms should respect the FAIR principle [87]. *brainlife.io* was built with the FAIR principles in mind and below, we pair each FAIR principle with the modern definition of neuroscience data. In the *brainlife.io* project, each principle is applied to multiple research assets, data derivatives, analysis software, and software services.

The three primary research assets pertaining to the *brainlife.io* project are (1) data, derivatives, and metadata, (2) processing applications and data analysis code, and (3) data and analysis management services are each made FAIR via the *brainlife.io* project.

Findable. Research data services available on brainlife.io such as data sets, processing App, web services and analysis code are either automatic or manual mechanisms to make them findable. brainlife.io assigns Digital-Objects-Identifiers (DOI) using DataCite as a partner project. DOIs are automatically assigned to publication records consisting of datasets, as well as versioned preprocessing and analysis software. These brainlife.io publication records are compliant with schema.org and as such are also compliant with Google Dataset search (https://datasetsearch.research.google.com). DOIs are also assigned to each published App.

Accessible. Data and metadata can be retrieved using a number of access methods via Web Interfaces and Command Line Interfaces. Metadata is also accessible programmatically via a web API. Metadata remains available even in the case that data must be removed (e.g., in cases of human subjects concerns). Authentication is necessary to access the data and users' identities are checked by humans to assure compliance with more restrictive data-access policies such as the GDPR. A full record of data management and processing is made accessible. So not just data or analysis streams are accessible but a full record reporting the provenance of each individual data product. The code underlying each processing App is accessible via GitHub, and can be modified or used via common GitHub mechanisms (push requests, pull requests). Previously published datasets can be downloaded to a local machine or copied to a new project.

Interoperable. Data can be submitted to *brainlife.io* either using standard file types such as NifTis, but also data from multiple vendors can be used to map the data to the BIDS standard and uploaded on the system using the brainlife.io/ezBIDS web tool. The brainlife.io/ezBIDS system allows data from multiple vendors and type of sequences to be mapped to the Brain Imaging Data Structure (BIDS) and from there to be pushed to *brainlife.io* Projects, to OpenNeuro.org or downloaded. Furthermore, datasets can be mapped from major archives and projects such as NKI, and OpenNeuro.org using DataLad.org. Finally, *brainlife.io* Apps on their own also use are FAIR, as they are publicly available both as services on brainlife.io and code implementing the services on GitHub. The Apps can be stored either on individual user or organization accounts or on the *brainlife.io* team GitHub account depending on the level of commitment of the app developer to maintaining the Apps. The *brainlife.io* team maintains a bl2bids (https://github.com/brainlife/abcd-spec/blob/master/hooks/bl2bids.py) and the BIDS Walked (https://github.com/brainlife/cli/blob/master/bids-walker.js) script that together allow mapping BIDS data types to *brainlife.io* DataTypes. As a result the BIDS standard is the data exchange approach used to increase data interoperability.

Reusable. The brainlife.io project has multiple aspects of its technology that is developed with a mindset focus of reuse. First, the whole platform is developed as open source and published on GitHub.com. Second, the data processing Applications are developed using a lightweight specification that is compatible with BIDS and can be easily used without brainlife.io interfaces on local computers or clusters. Finally, data assets can be shared within the platform across users and projects but also outside of the platform by downloading the data as BIDS-compliant datasets. Data derivatives, processing apps, and analysis notebooks can be accessed in multiple ways via web graphical user interfaces, command line interfaces, or directly via local download. Analysis notebooks in the form of Jupyter notebooks can be pushed to GitHub directly, allowing for instantaneous reuse by the broader community. Data pipelines can be copied and reused within a given project. All configuration parameters for each App are stored, allowing users to reuse previously defined optimal parameters for their given data. The *brainlife.io* publication modelis a key component to implementing a vision of an integrated project publication containing data, and preprocessing for future reuse.

Supplemental Table 1: Platform services serving the brainlife.io platform.

Service	Description	GitHub Repos
UI	Platform entrypoint, providing an user interface that integrates the diverse services in Brainlife	https://github.com/brainlife/warehouse/tree/master/ui
Warehouse	Data storage and management	https://github.com/brainlife/warehouse/
Amaretti	Automated scheduling servicer identifying appropriate compute resources and staging and archiving data	https://github.com/brainlife/amaretti/
ezBIDS	DICOM to BIDS conversion	https://github.com/brainlife/ezbids/
Vis	Services available for running visualizations within the platform	https://github.com/brainlife/brainlife/tree/master/vis
Event	Event-driven integrator, to provide real-time feedback for users	https://github.com/brainlife/event/
Service Monitoring	Monitors individual actions performed by the site	https://github.com/brainlife/servicemonitor
CLI	Command-line interface for performing data manipulations and data scrubbing	https://github.com/brainlife/cli
Auth	Centralized authentication for the multiple Brainlife services	https://github.com/brainlife/auth

Supplemental Table 1. Table with list of all platform services, name, scope, service URL (pointer to brainlife page if available as direct URL) and github URL for code.

Supplemental Table 2: Jupyter notebooks for analyses performed.

Notebook Name	Topic	Analysis/Figure	Datatype(s)	Measure(s)	Github URL
blp-analysis-structural-mri-volume.ipynb	Structural morphometry	Validity, reliability, reproducibility, development, references	neuro/parc-stats	Cortical parcel volume, thickness, surface area, Fractional Anisotropy (FA), Axial Diffusivity (AD), Radial Diffusivity (RD), Mean Diffusivity (MD), Neurite density index (NDI), Orientation dispersion index (ODI), Isotropic volume fraction (IsoVF)	https://github.com/bacaron/bp-notebooks/bl_paper/blp-analysis-structural-mri-volume.ipynb
blp-analysis-diffusion-mri-tract-profiles.ipynb	Diffusion profilometry	Validity, reliability, reproducibility, development, references	neuro/tractmeasures	White matter tract Fractional Anisotropy (FA), Axial Diffusivity (AD), Radial Diffusivity (RD), Mean Diffusivity (MD), Neurite density index (NDI), Orientation dispersion index (ODI), Isotropic	https://github.com/bacaron/bp-notebooks/bl_paper/blp-analysis-diffusion-mri-tract-profiles.ipynb

				volume fraction (IsoVF)	
blp-analysis-diffusion-mri-structural-connectivity.ipynb	Structural connectivity	Validity, reliability, reproducibility, development, references	neuro/network	Max node degree	https://github.com/bacaron/bp-notebooks/bl_paper/blp-analysis-diffusion-mri-structural-connectivity.ipynb
blp-analysis-functional-mri-functional-connectivity.ipynb	Functional connectivity	Validity, reliability, reproducibility, development, references	neuro/network	Within-network connectivity	https://github.com/bacaron/bp-notebooks/bl_paper/blp-analysis-functional-mri-functional-connectivity.ipynb
blp-analysis-functional-mri-gradienty.ipynb	Functional gradients	Validity, reliability, reproducibility, development, references	neuro/gradients	Distance of primary gradient	https://github.com/bacaron/bp-notebooks/bl_paper/blp-analysis-functional-mri-gradientsy.ipynb
blp-analysis-meeg-power-spectrum-density.ipynb	MEEG	Validity, reliability, reproducibility, development, references	neuro/meeg/psd	Peak alpha frequency, power spectrum density	https://github.com/bacaron/bp-notebooks/bl_paper/blp-analysis-meeg-power-spectrum-density.ipynb
blp-analysis-concussion-structural-mri.ipynb	Cortical diffusion	Clinical populations	neuro/parc-stats	Cortical parcel volume, thickness, surface area, Fractional Anisotropy (FA), Axial Diffusivity (AD), Radial Diffusivity (RD), Mean Diffusivity (MD), Neurite density index (NDI), Orientation dispersion index (ODI), Isotropic volume fraction (IsoVF)	https://github.com/bacaron/bp-notebooks/bl_paper/blp-analysis-concussion-structural-mri.ipynb
blp-analysis-inherited-retinal-disease.ipybn	Diffusion profilometry, optical coherence tomography (OCT)	Clinical populations	neuro/tractmeasures, neuro/microperimetry	White matter tract Fractional Anisotropy (FA), Photoreceptor thickness	https://github.com/bacaron/bp-notebooks/bl_paper/blp-analysis-inherited-retinal-disease.ipynb
blp-analysis-usage-statistics.ipynb	Platform usage statistics	NA	NA	NA	https://github.com/bacaron/bp-notebooks/bl_paper/blp-analysis-usage-statistics.ipynb

Supplemental Table 2. Description and web-links to the open-source code used for each analysis outlined previously in the form of individual Jupyter Notebooks.

Supplemental Table 3: Preprocessing Apps used for the experiments.

Name	Brainlife DOI	Github Repository
Anatomically Constrained Tractography using precomputed 5tt & CSD	10.25663/brainlife.app.297	bacaron/app-mrtrix3-act
mrtrix3 - WMC Anatomically Constrained Tractography (ACT)	10.25663/brainlife.app.319	brainlife/app-mrtrix3-act
Compile tract macro-structural and profile data	10.25663/brainlife.app.397	brainlife/app-compile-macro-micro-tract-stats
Compute summary statistics of diffusion measures from subcortical segmentation	10.25663/brainlife.app.389	brainlife/app-freesurfer-stats
Compute summary statistics of diffusion measures mapped to the cortical surface - Deprecated Surface	10.25663/brainlife.app.383	brainlife/app-cortex-tissue-mapping-stats
Conmat 2 Network	10.25663/brainlife.app.393	filipinascimento/bl-conmat2network
Convert network neuro matrix to conmat	10.25663/brainlife.app.335	brainlife/app-network-matrices-2-mat
Cortex Tissue Mapping (Native & Template Space)	10.25663/brainlife.app.379	brainlife/app-cortex-tissue-mapping
Fit Constrained Deconvolution Model for Tracking	10.25663/brainlife.app.238	bacaron/app-mrtrix3-act
Freesurfer	10.25663/bl.app.0	brainlife/app-freesurfer
Freesurfer Statistics	10.25663/brainlife.app.272	brainlife/app-freesurfer-stats
FSL Anat (T1)	10.25663/brainlife.app.273	brainlife/app-fsl-anat
Align T1 to ACPC Plane (HCP-based)	10.25663/bl.app.99	brainlife/app-hcp-acpc-alignment
FSL Anat (T2)	10.25663/brainlife.app.350	brainlife/app-fsl-anat
FSL Brain Extraction (BET) on DWI	10.25663/brainlife.app.163	brainlife/app-FSLBET
mrtrix3 preprocess	10.25663/bl.app.68	brainlife/validator-neuro-dwi
Multi-Atlas Transfer Tool (w/surface output)	10.25663/bl.app.23	faskowit/app-multiAtlasTT
Noddi Amico	10.25663/brainlife.app.365	brainlife/app-noddi-amico
Parcellation Statistics - Surface - Deprecated Datatype	10.25663/brainlife.app.464	brainlife/app-freesurfer-stats
Remove Tract Outliers	10.25663/brainlife.app.195	brainlife/validator-neuro-wmc
Tissue-type segmentation	10.25663/brainlife.app.239	brainlife/app-mrtrix3-5tt
Tract Analysis Profiles	10.25663/brainlife.app.361	brainlife/app-tractanalysisprofiles
Tractography quality check	10.25663/brainlife.app.189	brainlife/app-tractographyQualityCheck
White Matter Anatomy Segmentation	10.25663/brainlife.app.188	brainlife/validator-neuro-wmc
Align T2 to ACPC Plane (HCP-based)	10.25663/brainlife.app.116	brainlife/app-hcp-acpc-alignment/tree/1.4
fMRIPrep - Volume Output	10.25663/brainlife.app.160	brainlife/app-fmriprep/tree/20.2.3-2
pRFs / Benson14-Retinotopy - Deprecated	10.25663/brainlife.app.187	davhunt/app-benson14-retinotopy/tree/master
Segment thalamic nuclei	10.25663/brainlife.app.222	brainlife/app-segment-thalamic-nuclei/tree/v1.0
Track The Human Optic RAdiation (THORA): Contrack - Eccentricity	10.25663/brainlife.app.252	brainlife/app-contrack-optic-radiation/tree/v1.1
Automated Segmentation of Hippocampal Subfields (ASHS)	10.25663/brainlife.app.262	svincibo/app-ashs-segment/tree/master
fMRIPrep - Surface Output	10.25663/brainlife.app.267	brainlife/app-fmriprep/tree/20.2.1

FSL DTIFIT	10.25663/brainlife.app.292	brainlife/app-fslDTIFIT/tree/v1.1
fMRI Timeseries Extraction	10.25663/brainlife.app.369	faskowit/app-fmri-2-mat/tree/0.1.6
Structural Connectome MRTrix3 (SCMRT) - No labels or weights	10.25663/brainlife.app.395	brainlife/app-sift2-connectome-generation/tree/nosift2_v1.2_centers_netneuro
Generate Visual Regions of Interest Binned by Eccentricity Estimates (Benson Atlas) - Diffusion Space	10.25663/brainlife.app.414	brainlife/app-roiGenerator/tree/visual-white-matter-eccentricity-dwi-v1.2
dsi-studio-atk	10.25663/brainlife.app.423	frankyeh/dsi-studio-atk/tree/master
Apply Maxwell filter on MEG signals using MNE-python	10.25663/brainlife.app.476	brainlife/app-maxwell-filter/tree/master
Compute summary statistics of diffusion measures mapped to cortical surface	10.25663/brainlife.app.483	brainlife/app-cortex-tissue-mapping-stats/tree/updated-surface-dtype-v1.1
Split MEG file	10.25663/brainlife.app.529	guiomar/app-meg-split-fif/tree/main
PSD: Power Spectral Density (Welch method)	10.25663/brainlife.app.530	guiomar/app-psd/tree/main
Find frequency peak of PSD data	10.25663/brainlife.app.531	guiomar/app-peak-frequency/tree/master
Time series to network	10.25663/brainlife.app.532	filipinascimento/bl-timeseries2network/tree/0.2
Connectivity Gradients	10.25663/brainlife.app.574	anibalsolon/app-connectivity-gradient/tree/main
Average channels	10.25663/brainlife.app.599	guiomar/app-average-channels/tree/main

Supplemental Table 3. Description and web links to the open-source code and open cloud services used to perform the evaluation experiments described in the main article.

Supplementary Table 4. Validity and reliability correlation tables.

Modality	Measure	Analysis	Parcellation	r	rmse
Structural MRI	Cortical thickness	Validity	Destrieux	0.8667	0.2332
"	Cortical surface area	Validity	Destrieux	0.9774	173.9724
"	Cortical volume	Validity	Destrieux	0.9817	570.543
"	Cortical thickness	Reliability	Destrieux	0.9569	0.121
"	Cortical surface area	Reliability	Destrieux	0.9930	97.4636
"	Cortical volume	Reliability	Destrieux	0.9948	2378.1114
"	Cortical thickness	Validity	hcp-mmp	0.8449	0.2416
"	Cortical surface area	Validity	hcp-mmp	0.9835	78.1686
"	Cortical volume	Validity	hcp-mmp	0.9727	265.6
"	Cortical thickness	Reliability	hcp-mmp	0.9402	0.1394
"	Cortical surface area	Reliability	hcp-mmp	0.9952	41.7407
"	Cortical volume	Reliability	hcp-mmp	0.9933	123.118
Diffusion MRI	Tract AD	Validity	wma	0.9572	0.0309
"	Tract FA	Validity	wma	0.9515	0.0181
"	Tract MD	Validity	wma	0.9167	0.0200
"	Tract RD	Validity	wma	0.9817	0.0228
"	Tract AD	Reliability	wma	0.9204	0.0402
"	Tract FA	Reliability	wma	0.9312	0.0167
"	Tract MD	Reliability	wma	0.806	0.0292
"	Tract RD	Reliability	wma	0.8447	0.0282
Functional MRI	Node connectivity	Validity	Yeo17	0.8853	0.1219
"	Node connectivity	Reliability	Yeo17	0.7264	0.1889
"	Primary gradient	Validity	Shaffer400	0.5934	0.0358
"	Primary gradient	Reliability	Shaffer400	0.8496	0.0259
MEEG	Peak alpha frequency	Validity	NA	0.9385	0.2964
"	Peak alpha frequency	Reliability	NA	0.8484	0.4751

Supplemental Table 4. Pearson correlation (*r*) and root mean square error (*rmse*) for all validity and reliability experiments performed.

Supplemental Table 5: Resources for data storage, archiving, and computational analysis.

Location(s)	Archive Name	Web URL	Type	Archive Representative	Data Modality (-ies)	Type of access	Reference (publication)
U.S.A	BRAIN Initiative Cell Census Network (BICCN)	www.biccn.org/	service registry	Multiple; the Allen Institute has an NIH grant to build and host this site, through the Brain Cell Data Center (BCDC)	human, mouse; single cell RNA-Seq, Patch-Seq, cell morphologies, electrophysiological recordings (NWB files), multiple histological image modalities, mFISH		
US BRAIN	BICCN Single Cell Portal	singlecell.broadinstitute.org/single_cell	service registry	Broad Institute scp-support@broadinstitute.zendesk.com	Multiple single cell datasets	N/A	
US BRAIN	OpenNeuro.org	OpenNeuro.org	Archive	Russ Poldrack	human MRI, PET, EEG,		
US BRAIN	DABI archive	dabi.loni.usc.edu/home	Archive	TOGA, ARTHUR W	EEG, MEG, iEEG		
US BRAIN	Allen Brain Map	portal.brain-map.org	service registry	Allen Institute - multiple teams involved	human, mouse, rhesus macaque		
US BRAIN	DANDI	www.dandiarchive.org/	Archive	Satrajit Ghosh	Neurophysiology (EPhys, ICEphys, Ophys)		
US BRAIN	NeMO	nemoarchive.org/	Archive	Owen R. White	Multi-omics data		
US BRAIN	Brain Image Library (BIL)	www.brainimagelibrary.org/	service registry	ROPELEWSKI, ALEXANDER J	Brain imaging data		
US BRAIN	BossDB	bossdb.org/	Archive	WESTER, BROCK A.	EM		
US BRAIN	MiCRONS Explorer	microns-explorer.org/	web-service	Multiple	EM		
US BRAIN	[their main site]	www.braininitiative.org/resources/	service registry		aggregator		
US BRAIN	brainlife.io	brainlife.io	computational platforms	Franco Pestilli	MRI/EEG/MEG	Governed via license	
Australian Initiative		neurodesk.org	web-service				
Japan Initiative	SRPBS	www.cns.atr.jp/decnefpro/	service registry	Saori Tanaka, Mitsuo Kawato	Brain imaging data		
Japan Initiative	Brain/MINDS Beyond	mriportal.umin.jp/	service registry	Kiyoto Kasai, Takashi Hanakawa, Saori Tanaka	Brain imaging data		

Japan Initiative	Brain/MINDS	www.brainminds.riken.jp/	service registry	Alex Woodward	Marmoset atlas, fMRI, dMRI, tracer, gene expression	Open to collaborators	
China Initiative	Linked Brain Data	www.linked-brain-data.org/	service registry				
Korea Initiative	Korea Brain Initiative	kbrain-map.kbri.re.kr:8080/	service registry	Sung-Jin Jeong	mouse; single cell RNA-Seq, EM data (current); omics data, behavioural data, electrophysiology data (in future)		
European Human Brain Project	EBRAINS	ebrains.eu/	service registry	Jan Bjaalie	Brain imaging data, omics data, behavioural data, electrophysiology data, models etc	Closed	
Canadian Open Neuroscience Platform	CONP	conp.ca/	service registry	CONP committee	Brain imaging data, omics data, behavioural data, electrophysiology data, models etc	Governed via license	
BlueBrainProject		channelpedia.epfl.ch/	service registry				
DataLad		datasets.datalad.org/	service registry			Fully open (CC-00)	
NITRC			service registry				
USA	WebPlotDigitizer	automeris.io/WebPlotDigitizer/	web-service	Ankit Rohatgi			
USA	Brain Map Database	brainmap.org	web-service	Peter Fox	Brain Imaging data	Governed via license	
USA	NeuroSynth Database	neurosnyth.org	web-service	Alejandro de la Vega	Brain Imaging data	Fully open (CC-00)	
France	NeuroQuery	https://neuroquery.org	web-service	INRIA/ Jérôme Dockès	Brain Imaging data	Fully open (CC-00)	
	OSF	osf.io	Archive		Unspecified / Open	Unspecified	
U.S.A.	COINSTAC	https://coinstac.org/	Downloadable	Georgia State University	Brain Imaging Data	Unspecified	

Supplemental Table 5. Description and web links to the many available platforms and services for increasing data gravity in the neuroimaging field.

Supplemental Table 6: Processed dataset published as part of this article.

Project	DOI	Brainlife Publication URL
Human Connectome Young Adult - Test - Retest	https://doi.org/10.25663/brainlife.pub.38	https://brainlife.io/pub/640a3da8c538c16a826f912e
Human Connectome Young Adult - Full Dataset	https://doi.org/10.25663/brainlife.pub.40	https://brainlife.io/pub/640a3f9dc538c16a826f9b1a
Cambridge Centre for Ageing and Neuroscience - Full Dataset	https://doi.org/10.25663/brainlife.pub.39	https://brainlife.io/pub/640a3f0cc538c16a826f9648
MEG [fif] Cam-Can	https://doi.org/10.25663/brainlife.pub.41	https://brainlife.io/pub/640a40fec538c16a826fa468
MEG [fif] Run1 vs Run2	https://doi.org/10.25663/brainlife.pub.42	https://brainlife.io/pub/640a4155c538c16a826fa5b9
MEG [fif] CamCan-maxfilt	https://doi.org/10.25663/brainlife.pub.43	https://brainlife.io/pub/640a41abc538c16a826fa6e6
ASHS Segmentation of Hippocampal Subfields - Replication derivatives	https://doi.org/10.25663/brainlife.pub.44	https://brainlife.io/pub/640a4267c538c16a826fb09a

Supplemental Table 1. Table with list of all platform services, name, scope, service URL (pointer to brainlife page if available as direct URL) and github URL for code.

REFERENCES

1. Stewart, C. A. *et al.* Jetstream: A self-provisioned, scalable science and engineering cloud environment. (2015) doi:10.1145/2792745.2792774.
2. Hancock, D. Y. *et al.* Jetstream2: Accelerating cloud computing via Jetstream. in *Practice and Experience in Advanced Research Computing* 1–8 (Association for Computing Machinery, 2021). doi:10.1145/3437359.3465565.
3. Dale, A., Fischl, B. & Sereno, M. I. Cortical Surface-Based Analysis: I. Segmentation and Surface Reconstruction. *Neuroimage* **9**, 179–194 (1999).
4. Fischl, B., Sereno, M. I. & Dale, A. Cortical Surface-Based Analysis: II: Inflation, Flattening, and a Surface-Based Coordinate System. *Neuroimage* **9**, 195–207 (1999).
5. Desikan, R. S. *et al.* An automated labeling system for subdividing the human cerebral cortex on MRI scans into gyral based regions of interest. *Neuroimage* **31**, 968–980 (2006).
6. Fischl, B., Liu, A. & Dale, A. M. Automated manifold surgery: constructing geometrically accurate and topologically correct models of the human cerebral cortex. *IEEE Medical Imaging* **20**, 70–80 (2001).
7. Fischl, B. *et al.* Whole brain segmentation: automated labeling of neuroanatomical structures in the human brain. *Neuron* **33**, 341–355 (2002).
8. Fischl, B. *et al.* Sequence-independent segmentation of magnetic resonance images. *Neuroimage* **23**, S69–S84 (2004).
9. Fischl, B., Sereno, M. I., Tootell, R. B. H. & Dale, A. M. High-resolution intersubject averaging and a coordinate system for the cortical surface. *Hum. Brain Mapp.* **8**, 272–284 (1999).
10. Fischl, B. *et al.* Automatically Parcellating the Human Cerebral Cortex. *Cereb. Cortex* **14**, 11–22 (2004).
11. Han, X. *et al.* Reliability of MRI-derived measurements of human cerebral cortical thickness: The effects of field strength, scanner upgrade and manufacturer. *Neuroimage* **32**, 180–194 (2006).
12. Jovicich, J. *et al.* Reliability in multi-site structural MRI studies: Effects of gradient non-linearity correction on phantom and human data. *Neuroimage* **30**, 436–443 (2006).
13. Kuperberg, G. R. *et al.* Regionally localized thinning of the cerebral cortex in Schizophrenia. *Arch. Gen. Psychiatry* **60**, 878–888 (2003).

14. Reuter, M., Schmansky, N. J., Rosas, H. D. & Fischl, B. Within-Subject Template Estimation for Unbiased Longitudinal Image Analysis. *Neuroimage* **61**, 1402–1418 (2012).
15. Reuter, M. & Fischl, B. Avoiding Asymmetry-Induced Bias in Longitudinal Image Processing. *Neuroimage* **57**, 19–21 (2011).
16. Reuter, M., Rosas, H. D. & Fischl, B. Highly Accurate Inverse Consistent Registration: A Robust Approach. *Neuroimage* **53**, 1181–1196 (2010).
17. Salat, D. *et al.* Thinning of the cerebral cortex in aging. *Cereb. Cortex* **14**, 721–730 (2004).
18. Segonne, F. *et al.* A hybrid approach to the skull stripping problem in MRI. *Neuroimage* **22**, 1060–1075 (2004).
19. Segonne, F., Pacheco, J. & Fischl, B. Geometrically accurate topology-correction of cortical surfaces using nonseparating loops. *IEEE Trans. Med. Imaging* **26**, 518–529 (2007).
20. Tournier, J.-D., Calamante, F. & Connelly, A. MRtrix: Diffusion tractography in crossing fiber regions. *Int. J. Imaging Syst. Technol.* **22**, 53–66 (2012).
21. Tournier, J.-D. *et al.* MRtrix3: A fast, flexible and open software framework for medical image processing and visualisation. *Neuroimage* **202**, 116137 (2019).
22. Esteban, O. *et al.* fMRIPrep: a robust preprocessing pipeline for functional MRI. *Nat. Methods* **16**, 111–116 (2019).
23. Cieslak, M. *et al.* QSIPrep: an integrative platform for preprocessing and reconstructing diffusion MRI data. *Nat. Methods* **18**, 775–778 (2021).
24. Developers, S. *SingularityCE 3.8.3*. (2021). doi:10.5281/zenodo.5564915.
25. Merkel, D. Docker: lightweight Linux containers for consistent development and deployment. *Linux J.* **2014**, 2 (2014).
26. DataCite Schema. *DataCite Schema* https://schema.datacite.org/meta/kernel-4.1/index.html.
27. Gonzalez-Beltran, A. N. *et al.* Data discovery with DATS: exemplar adoptions and lessons learned. *J. Am. Med. Inform. Assoc.* **25**, 13–16 (2018).
28. Gonzalez-Beltran, A. & Rocca-Serra, P. *biocaddie/WG3-MetadataSpecifications: DataMed DATS specification v2.2 - NIH BD2K bioCADDIE*. (2017). doi:10.5281/zenodo.438337.

29. Caron, B. *et al.* Collegiate athlete brain data for white matter mapping and network neuroscience. *Sci Data* **8**, 56 (2021).

30. McPherson, B. C. & Pestilli, F. A single mode of population covariation associates brain networks structure and behavior and predicts individual subjects' age. *Commun Biol* **4**, 943 (2021).

31. Bertò, G. *et al.* Classifyber, a robust streamline-based linear classifier for white matter bundle segmentation. *Neuroimage* **224**, 117402 (2021).

32. Sharmin, N., Olivetti, E. & Avesani, P. White Matter Tract Segmentation as Multiple Linear Assignment Problems. *Front. Neurosci.* **11**, 754 (2017).

33. Vinci-Booher, S., Caron, B., Bullock, D., James, K. & Pestilli, F. Development of white matter tracts between and within the dorsal and ventral streams. *Brain Struct. Funct.* **227**, 1457–1477 (2022).

34. Kurzawski, J. W., Mikellidou, K., Morrone, M. C. & Pestilli, F. The visual white matter connecting human area prostriata and the thalamus is retinotopically organized. *Brain Struct. Funct.* **225**, 1839–1853 (2020).

35. Sani, I. *et al.* The human endogenous attentional control network includes a ventro-temporal cortical node. *Nat. Commun.* **12**, 360 (2021).

36. Allen, E. J. *et al.* A massive 7T fMRI dataset to bridge cognitive neuroscience and artificial intelligence. *Nat. Neurosci.* **25**, 116–126 (2022).

37. Puzniak, R. J. *et al.* CHIASM, the human brain albinism and achiasma MRI dataset. *Sci Data* **8**, 308 (2021).

38. Hanekamp, S. *et al.* White matter alterations in glaucoma and monocular blindness differ outside the visual system. *Sci. Rep.* **11**, 6866 (2021).

39. Cheng, H. *et al.* Denoising diffusion weighted imaging data using convolutional neural networks. *PLoS One* **17**, e0274396 (2022).

40. Eke, D. O. *et al.* International data governance for neuroscience. *Neuron* (2021) doi:10.1016/j.neuron.2021.11.017.

41. Gorgolewski, K. J. *et al.* The brain imaging data structure, a format for organizing and describing outputs of neuroimaging experiments. *Sci Data* **3**, 160044 (2016).

42. Halchenko, Y. *et al.* DataLad: distributed system for joint management of code, data, and their relationship. *J. Open Source Softw.* **6**, 3262 (2021).

43. Markiewicz, C. J. *et al.* The OpenNeuro resource for sharing of neuroscience data. *Elife* **10**, (2021).

44. Nooner, K. B. *et al.* The NKI-Rockland Sample: A Model for Accelerating the Pace of Discovery Science in Psychiatry. *Front. Neurosci.* **6**, 152 (2012).

45. Tobe, R. H. *et al.* A longitudinal resource for studying connectome development and its psychiatric associations during childhood. *Sci Data* **9**, 300 (2022).

46. Di Martino, A. *et al.* The autism brain imaging data exchange: towards a large-scale evaluation of the intrinsic brain architecture in autism. *Mol. Psychiatry* **19**, 659–667 (2014).

47. Dean, J. & Ghemawat, S. MapReduce: simplified data processing on large clusters. *Commun. ACM* **51**, 107–113 (2008).

48. Kluyver, T. *et al.* Jupyter Notebooks – a publishing format for reproducible computational workflows. in *Positioning and Power in Academic Publishing: Players, Agents and Agendas* (eds. Loizides, F. & Scmidt, B.) 87–90 (IOS Press, 2016). doi: Kluyver, Thomas, Ragan-Kelley, Benjamin, Pérez, Fernando, Granger, Brian, Bussonnier, Matthias, Frederic, Jonathan, Kelley, Kyle, Hamrick, Jessica, Grout, Jason, Corlay, Sylvain, Ivanov, Paul, Avila, Damián, Abdalla, Safia, Willing, Carol and Jupyter development team, (2016) Jupyter Notebooks – a publishing format for reproducible computational workflows. Loizides, Fernando and Scmidt, Birgit (eds.) In Positioning and Power in Academic Publishing: Players, Agents and Agendas. IOS Press. pp. 87-90 . (doi:10.3233/978-1-61499-649-1-87 <http://dx.doi.org/10.3233/978-1-61499-649-1-87>). .

49. Perez, F. & Granger, B. E. IPython: A System for Interactive Scientific Computing. *Comput. Sci. Eng.* **9**, 21–29 (2007).

50. Wickham, H. Tidy Data. *J. Stat. Softw.* **59**, 1–23 (2014).

51. Avesani, P. *et al.* The open diffusion data derivatives, brain data upcycling via integrated publishing of derivatives and reproducible open cloud services. *Sci Data* **6**, 69 (2019).

52. Alexander, L. M. *et al.* An open resource for transdiagnostic research in pediatric mental health and learning disorders. *Sci Data* **4**, 170181 (2017).

53. National Academies of Sciences, Engineering *et al. Understanding Reproducibility and Replicability*. (National Academies Press (US), 2019).

54. Kelley, T. L. Interpretation of educational measurements. **353**, (1927).

55. Van Essen, D. C. *et al.* The WU-Minn Human Connectome Project: an overview. *Neuroimage* **80**, 62–79 (2013).
56. Shafto, M. A. *et al.* The Cambridge Centre for Ageing and Neuroscience (Cam-CAN) study protocol: a cross-sectional, lifespan, multidisciplinary examination of healthy cognitive ageing. *BMC Neurol.* **14**, 204 (2014).
57. Casey, B. J. *et al.* The Adolescent Brain Cognitive Development (ABCD) study: Imaging acquisition across 21 sites. *Dev. Cogn. Neurosci.* **32**, 43–54 (2018).
58. Yeo, B. T. T. *et al.* The organization of the human cerebral cortex estimated by intrinsic functional connectivity. *J. Neurophysiol.* **106**, 1125–1165 (2011).
59. Bethlehem, R. A. I. *et al.* Dispersion of functional gradients across the adult lifespan. *Neuroimage* **222**, 117299 (2020).
60. Margulies, D. S. *et al.* Situating the default-mode network along a principal gradient of macroscale cortical organization. *Proc. Natl. Acad. Sci. U. S. A.* **113**, 12574–12579 (2016).
61. Taulu, S. & Kajola, M. Presentation of electromagnetic multichannel data: The signal space separation method. *J. Appl. Phys.* **97**, 124905 (2005).
62. Taulu, S. & Simola, J. Spatiotemporal signal space separation method for rejecting nearby interference in MEG measurements. *Phys. Med. Biol.* **51**, 1759–1768 (2006).
63. Pedregosa, F. *et al.* Scikit-learn: Machine Learning in Python. *J. Mach. Learn. Res.* **12**, 2825–2830 (2011).
64. Fukutomi, H. *et al.* Neurite imaging reveals microstructural variations in human cerebral cortical gray matter. *Neuroimage* **182**, 488–499 (2018).
65. Ho, T. C. *et al.* Effects of sensitivity to life stress on uncinate fasciculus segments in early adolescence. *Soc. Cogn. Affect. Neurosci.* **12**, 1460–1469 (2017).
66. Hanson, J. L., Knodt, A. R., Brigidi, B. D. & Hariri, A. R. Lower structural integrity of the uncinate fasciculus is associated with a history of child maltreatment and future psychological vulnerability to stress. *Dev. Psychopathol.* **27**, 1611–1619 (2015).
67. Yushkevich, P. A. *et al.* Automated volumetry and regional thickness analysis of hippocampal subfields and medial temporal cortical structures in mild cognitive impairment. *Hum. Brain Mapp.* **36**, 258–287 (2015).

68. Karcher, N. R. & Barch, D. M. The ABCD study: understanding the development of risk for mental and physical health outcomes. *Neuropsychopharmacology* **46**, 131–142 (2021).
69. McKee, A. C., Daneshvar, D. H., Alvarez, V. E. & Stein, T. D. The neuropathology of sport. *Acta Neuropathol.* **127**, 29–51 (2014).
70. Glasser, M. F. *et al.* A multi-modal parcellation of human cerebral cortex. *Nature* **536**, 171–178 (2016).
71. Yoshimine, S. *et al.* Age-related macular degeneration affects the optic radiation white matter projecting to locations of retinal damage. *Brain Struct. Funct.* **223**, 3889–3900 (2018).
72. Ogawa, S. *et al.* White matter consequences of retinal receptor and ganglion cell damage. *Invest. Ophthalmol. Vis. Sci.* **55**, 6976–6986 (2014).
73. Malania, M., Konrad, J., Jägle, H., Werner, J. S. & Greenlee, M. W. Compromised Integrity of Central Visual Pathways in Patients With Macular Degeneration. *Invest. Ophthalmol. Vis. Sci.* **58**, 2939–2947 (2017).
74. Sherbondy, A. J., Dougherty, R. F., Ben-Shachar, M., Napel, S. & Wandell, B. A. ConTrack: finding the most likely pathways between brain regions using diffusion tractography. *J. Vis.* **8**, 15.1–16 (2008).
75. Benson, N. C. *et al.* The retinotopic organization of striate cortex is well predicted by surface topology. *Curr. Biol.* **22**, 2081–2085 (2012).
76. Benson, N. C., Butt, O. H., Brainard, D. H. & Aguirre, G. K. Correction of distortion in flattened representations of the cortical surface allows prediction of V1-V3 functional organization from anatomy. *PLoS Comput. Biol.* **10**, e1003538 (2014).
77. Yeatman, J. D., Dougherty, R. F., Myall, N. J., Wandell, B. A. & Feldman, H. M. Tract profiles of white matter properties: automating fiber-tract quantification. *PLoS One* **7**, e49790 (2012).
78. Aydogan, D. B. & Shi, Y. Parallel Transport Tractography. *IEEE Trans. Med. Imaging* **40**, 635–647 (2021).
79. Baran, D. & Shi, Y. A novel fiber-tracking algorithm using parallel transport frames. in *ISMRM* (unknown, 2019).
80. Esteban, O. *et al.* MRIQC: Advancing the automatic prediction of image quality in MRI from unseen sites. *PLoS One* **12**, e0184661 (2017).
81. Yeh, F.-C. Shape analysis of the human association pathways. *Neuroimage* **223**, 117329 (2020).
82. Cameron, C. *et al.* Towards automated analysis of connectomes: The configurable pipeline for the analysis of

connectomes (C-PAC). *Front. Neuroinform.* **7**, (2013).

83. Fischl, B. FreeSurfer. *Neuroimage* **62**, 774–781 (2012).

84. Yanni, S. E. *et al.* Normative reference ranges for the retinal nerve fiber layer, macula, and retinal layer thicknesses in children. *Am. J. Ophthalmol.* **155**, 354–360.e1 (2013).

85. Keshavan, A., Yeatman, J. D. & Rokem, A. Combining Citizen Science and Deep Learning to Amplify Expertise in Neuroimaging. *Front. Neuroinform.* **13**, 29 (2019).

86. Infrastructure cards. https://www.incf.org/infrastructure-portfolio.

87. Sandström, M. *et al.* Recommendations for repositories and scientific gateways from a neuroscience perspective. *Sci Data* **9**, 212 (2022).
